# Formulating Smart All‐in‐One Chitosan Hydrogel for High Performance Wound Dressing

**DOI:** 10.1002/adhm.202502971

**Published:** 2025-10-06

**Authors:** Chia‐Chi Lin, Andi Magattang Gafur Muchlis, Ren‐Jei Chung, Ssu Yu Huang, Michal Martinka, Syang‐Peng Rwei, Aivaras Kareiva, Jen‐Chang Yang, Chun Che Lin

**Affiliations:** ^1^ Institute of Organic and Polymeric Materials National Taipei University of Technology No. 1, Section 3, Zhongxiao E. Rd, Daan District Taipei 10608 Taiwan; ^2^ Research and Development Center for Smart Textile Technology National Taipei University of Technology No. 1, Section 3, Zhongxiao E. Rd, Daan District Taipei 10608 Taiwan; ^3^ Department of Chemical Engineering and Biotechnology National Taipei University of Technology No. 1, Section 3, Zhongxiao E. Rd, Daan District Taipei 10608 Taiwan; ^4^ Industrial Technology Research Institute No. 321, Sec. 2, Guangfu Rd., East Dist. Hsinchu City 300044 Taiwan; ^5^ Department of Clothing Technology Technical University of Liberec Studentská 1402/2, Liberec 1 Liberec 461 17 Czech Republic; ^6^ Institute of Chemistry Vilnius University 3 Universiteto St., LT‐01513 Vilnius 03225 Lithuania; ^7^ Graduate Institute of Nanomedicine and Medical Engineering Taipei Medical University No.301, Yuantong Rd., Zhonghe Dist. New Taipei City 235 Taiwan

**Keywords:** antibacterial, chitosan hydrogel, hydrogen bond, thermosensitive, wound dressing

## Abstract

The poor mechanical and film formation properties of chitosan hydrogel limit its application as a wound dressing. To solve this shortcoming, chitosan‐graft‐poly(N‐isopropyl acrylamide) (PNIPAAm) and crosslinked with polyvinyl alcohol/polyvinyl pyrollidone (PVA/PVP) blend polymer is designed to form the thermosensitive hydrogel. After that, silver nanoparticles (AgNPs), synthesized by the co‐reduction method, are added into the chitosan hydrogel network to provide better antibacterial ability. When the temperature is above 32 °C, the hydrogel showed a decrease in particle size and transmittance, which proved that it is a thermal responsive material. Chitosan hydrogels are evaluated for their thermal properties, mechanical properties, swelling effects, antibacterial properties, and wound healing ability in rats. The results show that the hydrogel has a large number of intermolecular and intramolecular hydrogen bonds and a porous 3D network structure. The swelling ratio is 358.86 ± 23.56% and the degradation ratio is 86.02 ± 2.82% at 21 days. It also has excellent antibacterial properties against *Escherichia coli* and a much faster wound recovery ability. Therefore, a chitosan hydrogel with excellent physical properties is formulated in this study and has great potential as a smart all‐in‐one wound dressing for accelerating wound healing.

## Graphical abstract

An innovative approach to fabricating a thermosensitive, highly swelling, degradable, and antibacterial hydrogel composed of a chitosan‐g‐NIPAAm/PVA/PVP copolymer, integrated with AgNPs, for use in wound dressing applications. The hydrogel demonstrates enhanced performance, accelerating the wound healing process.

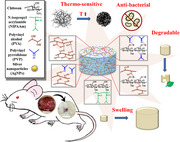

## 1 Introduction

Effective wound care remains a clinical challenge due to the complex healing process and risk of infection. Conventional wound dressings often fail to provide a moist healing environment, lack antibacterial properties, and do not adapt well to dynamic physiological conditions, which can delay recovery and increase patient discomfort.^[^ ^1^, ^2^ ^]^ Therefore, advanced wound dressing systems that are stimulus‐responsive, biocompatible, antibacterial, and mechanically stable are highly desirable.^[^ ^3^ ^]^

Hydrogels have emerged as promising candidates for biomedical applications due to their high water retention, tissue‐mimicking properties, and responsiveness to environmental stimuli such as temperature, pH, magnetic fields, and light.^[^ ^4^, ^5^, ^6^, ^7^, ^8^, ^9^, ^10^, ^11^, ^12^, ^13^, ^14^, ^15^, ^16^, ^17^ ^]^ Despite these advantages, hydrogels composed of a single component often exhibit poor mechanical strength and limited functional performance.^[^ ^18^ ^]^ These shortcomings restrict their effectiveness in applications like wound dressings, where durability and antibacterial activity are essential.

To address these limitations, we developed a multifunctional hydrogel system by integrating several advantageous components. First, we chose chitosan as the base material for the hydrogel. Chitosan is a biodegradable, biocompatible, and antibacterial natural polymer with multifunctional properties. Due to its abundance of amino groups, it exhibits high chemical reactivity.^[^ ^6^ ^]^ Its hemostatic properties have made it well known in the field of wound treatment.^[^ ^19^ ^]^ Chitosan not only promotes collagen fiber proliferation and accelerates wound healing, but also helps reduce scarring and preserve the skin's original appearance. However, chitosan hydrogels suffer from poor mechanical strength and film‐forming ability, which limits their effectiveness as wound dressings.^[^ ^20^ ^]^

To overcome the limitations of chitosan hydrogels in terms of mechanical strength and film‐forming ability, we incorporated poly(N‐isopropylacrylamide) (PNIPAAm) to impart smart, responsive functionality. PNIPAAm is a temperature‐sensitive polymer with a lower critical solution temperature (LCST) of ≈32 °C. When the ambient temperature is below the LCST, PNIPAAm is water‐soluble and exhibits a swollen, hydrophilic state. Above this temperature, the polymer transitions into a shrunken, hydrophobic state. This thermoresponsive behavior results from the interplay between hydrophobic interactions among isopropyl groups and hydrogen bonding within hydrophilic segments of the polymer. Notably, this transition is reversible with temperature changes.^[^ ^21^ ^]^ Due to its biostability and biocompatibility, PNIPAAm is highly attractive for smart biomedical applications, including wound dressings.^[^ ^11^ ^]^

To further enhance the mechanical and film‐forming properties of the hydrogel, polyvinyl alcohol (PVA) and polyvinylpyrrolidone (PVP) were incorporated. PVA is a semi‐crystalline polymer known for its ease of preparation and excellent chemical and physical resistance.^[^ ^22^ ^]^ It has been widely applied in biomedicine, including artificial pancreas systems, wound dressings, artificial skin, cardiovascular devices, etc.^[^ ^18^, ^23^ ^]^ However, its highly hydrophilic nature results in poor water stability, which limits its standalone application. To address this, PVA is often modified through copolymerization, grafting, or blending with other polymers.^[^ ^18^, ^24^ ^]^

PVP, on the other hand, is a high‐performance polymer with favorable properties such as biocompatibility, non‐toxicity, strong film‐forming ability, colloidal protection, chemical stability, and excellent solubility in both water and various organic solvents. It exhibits a balance of hydrophilic and hydrophobic characteristics, as well as acid and thermal stability, making it well‐suited for biomedical applications.^[^ ^25^, ^26^, ^27^ ^]^ However, like PVA, PVP has limited mechanical strength, and thus its performance can be significantly improved through blending with complementary polymers.^[^ ^28^ ^]^

To endow the hydrogel with antibacterial functionality—an essential feature for preventing wound infections—silver nanoparticles (AgNPs) were incorporated into the system. AgNPs are well‐known for their broad‐spectrum and potent antibacterial activity, primarily attributed to the controlled release of silver ions Ag^+^ ions, which can disrupt bacterial cell membranes, interfere with protein synthesis, and induce oxidative stress, ultimately leading to cell death. Their integration into the hydrogel not only enhances its antimicrobial performance but also contributes to maintaining a sterile wound environment throughout the healing process.^[^ ^29^, ^30^, ^31^ ^]^

In this study, chitosan was grafted with PNIPAAm using ammonium persulfate (APS) as the initiator and N,N,N“,N”‐tetramethylethylenediamine (TEMED) as the accelerator, then crosslinked with a PVA/PVP blend to form a robust hydrogel matrix. AgNPs were synthesized via a pH‐ and temperature‐controlled co‐reduction method to ensure stability and were uniformly embedded within the matrix. The resulting hydrogel was characterized using proton nuclear magnetic resonance (^1^H NMR), Fourier transform infrared (FT‐IR), scanning electron microscopy (SEM), and thermographimetric analysis (TGA). Its potential as a wound dressing was evaluated through mechanical testing, swelling behavior, degradation rate, cytotoxicity, antibacterial activity, and in vivo wound healing studies. This multifunctional hydrogel exhibits excellent thermoresponsiveness, biodegradability, high swelling capacity, and strong antibacterial properties, highlighting its promise for next‐generation wound dressing applications.

## 2 Results and Discussion

### 2.1 Synthesis and Characterization of the Chitosan‐g‐NIPAAm/PVA/PVP Hydrogels

**Scheme 1:**
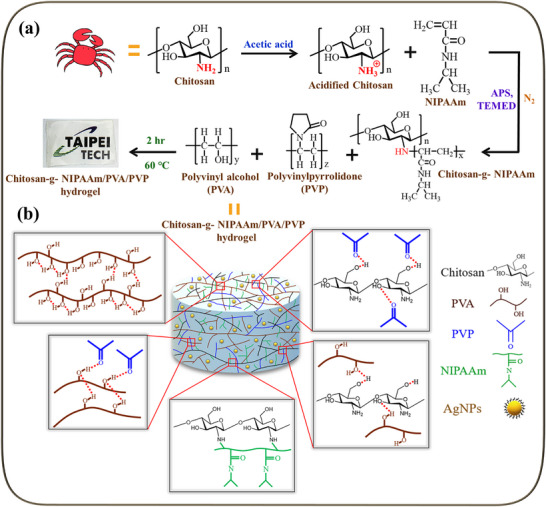
Schematic representation of the formation of chitosan hydrogels. a) Flow chart of synthesis of chitosan hydrogel; b) Schematic diagram of chitosan hydrogel structure.

The preparation of chitosan hydrogel is proposed as in **Scheme** **1**. Hydrogels were synthesized by a simple experimental method. They were formed by intermolecular hydrogen bonds between PVA and PVP, intermolecular and intramolecular hydrogen bonds between PVP and chitosan, intermolecular hydrogen bonds between PVA and chitosan, intramolecular hydrogen bonds between chitosan and chitosan, and chitosan grafted with NIPAAm. In this study, chitosan:NIPAAm ratio in grafted Chitosan‐g‐NIPAAm copolymer was regulated as 2:1, 1:1, and 1:2 named as C2N1, C1N1, and C1N2. On the other hand, the copolymer without NIPAAm grafting (Chitosan/PVA/PVP) was named CSPP in this paper. As shown in Table S1 (Supporting Information), the crosslinked copolymer appears pale yellow and can form a hydrogel through NaOH with a highly transparent film (the laboratory logo in the background). The copolymer containing AgNPs appears yellowish. A series of subsequent tests on the hydrogel proved that it was a suitable hydrogel for wound dressing.

**Figure 1:**
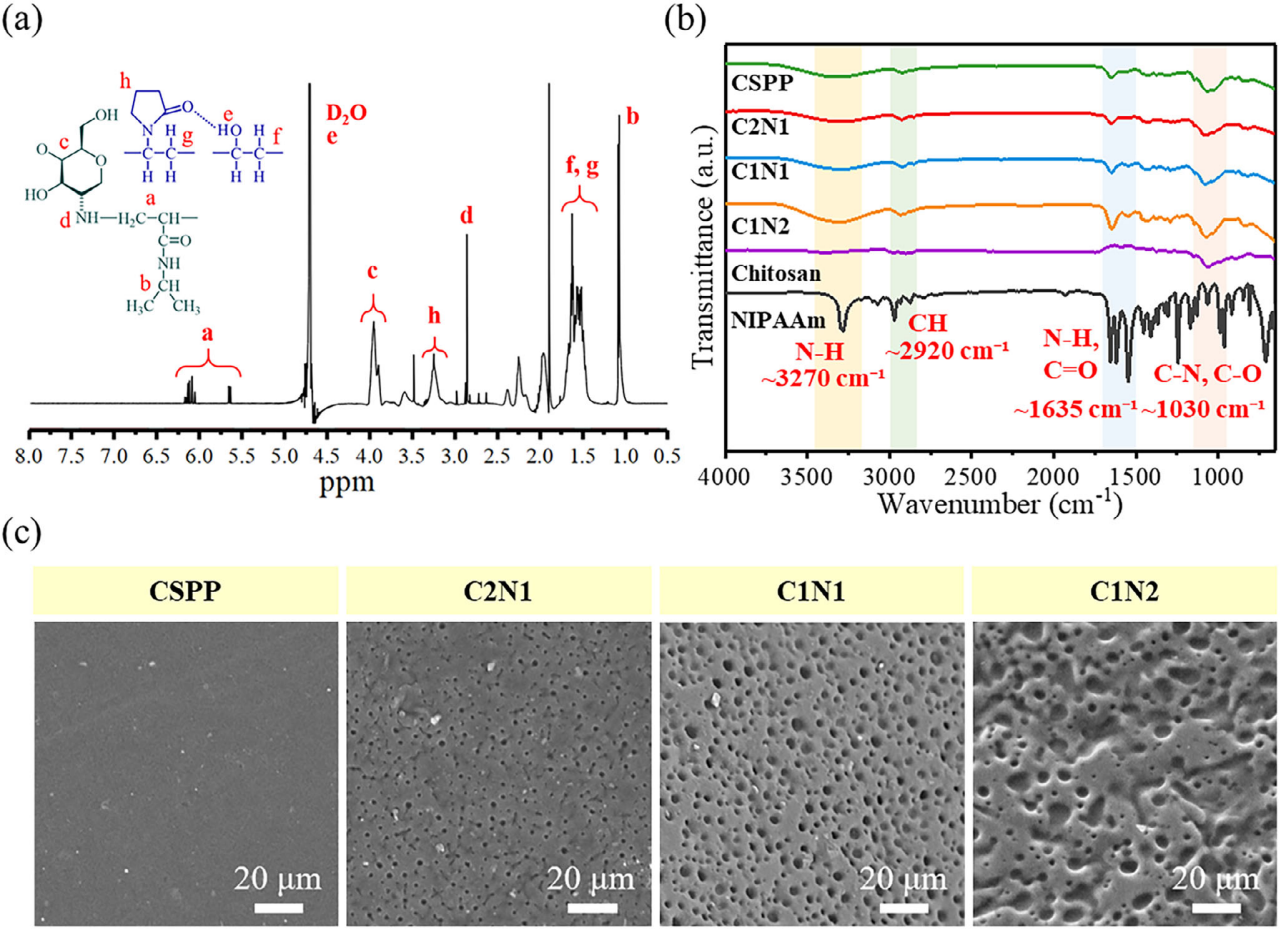
Characterization of hydrogels. a) ^1^H NMR of chitosan‐g‐NIPAAm hydrogel; b) FT‐IR spectra of hydrogels and monomer; c) SEM images of hydrogels, scale bar: 20 µm.

The chemical structure of chitosan‐g‐NIPAAm hydrogel was confirmed by ^1^H NMR, as shown in **Figure** **1**a. The ^1^H NMR spectrum of the monomer structure is shown in Figure S1 (Supporting Information). δ_a_ is the hydrogen of the CH_2_‐C and C‐CH groups on NIPAAm.^[^ ^32^ ^]^ δ_e_ is the peak of hydrogen on the OH group on PVA and D_2_O.^[^ ^33^ ^]^ δ_c_ is the hydrogen on the C‐CH‐C group on the six‐membered loop of chitosan. δ_h_ is hydrogen on the γ ‐lactamide CH group of PVP.^[^ ^34^ ^]^ δ_d_ is the hydrogen of the CH group adjacent to the primary amine on chitosan.^[^ ^35^ ^]^ δ_f,g_ are hydrogen signals of PVA and PVP C‐CH_2_ groups.^[^ ^33^ ^]^ δ_b_ is a hydrogen signal from the C‐CH_3_ group on NIPAAm.^[^ ^32^ ^]^ In addition, we have included the ^1^H NMR data of all prepared samples in Figure S2 (Supporting Information) to highlight the contrast between the non‐NIPAAm‐grafted polymer and the NIPAAm‐grafted polymers. Based on these results, we believe that the grafting process of chitosan with NIPAAm was successful.

The identification of specific functional groups in hydrogels has been performed by FT‐IR spectroscopy. As shown in Figure 1b, the broadband peaks between 3500 and 3250 cm^−1^ are due to the NH stretching vibration and the OH stretching of the intermolecular and intramolecular hydrogen bonds^[^ ^36^ ^]^ that may come from chitosan, NIPAAm, and PVA/PVP. The peak at 2950–2800 cm^−1^ was a typical C─H stretching vibration.^[^ ^27^ ^]^ The NH stretching vibration and C═O stretching vibration between 1700 and 1500 cm^−1^.^[^ ^37^ ^]^ In the range of 1068–1020 cm^−1^ is the signal of C─O stretching vibration and C─N stretching vibration overlap.^[^ ^38^ ^]^ All copolymer samples, such as C2N1, C1N1, and C1N2, show the absorbance band that are a combination of chitosan and NIPAAm. In addition, we can observe that with a bigger ratio of NIPAAm (C1N2) in the copolymer, the band at peaks of 3500–3250 cm^−1^, 2950–2800 cm^−1^, 1700–1500 cm^−1^, and 1068–1020 cm^−1^ were relatively stronger, following the characteristic of NIPAAm.

In this study, the surface morphology of the chitosan hydrogel was evaluated by SEM. As shown in Figure 1c, the surface of the chitosan‐g‐NIPAAm hydrogel series showed porous structures in line with commonly reported NIPAAm hydrogel.^[^ ^39^ ^]^ The circular holes on the surface of the C2N1 hydrogel are ≈2.25 ± 0.34 µm. The circular holes on the surface of the C1N1 hydrogel are ≈2.81 ± 0.64 µm. The surface of C1N2 hydrogel has irregular pores, with a size of ≈3.71 ± 1.41 µm. Compared to the control sample without NIPAAm (CSPP), which exhibits a smooth and compact surface under identical drying conditions, the more NIPAAm added, the larger the pores formed by the hydrogel. This trend suggests that NIPAAm contributes significantly to the development of the porous morphology. While we acknowledge that freeze‐drying can introduce structural artifacts, the consistent absence of pores in the non‐NIPAAm sample (CSPP), despite undergoing the same processing, indicates that the observed porosity is not solely a drying artifact. Instead, it likely arises from the hydrophilic amide groups in NIPAAm, which can form hydrogen bonds with water in the hydrated state. Upon drying, the removal of these water molecules may result in voids, leading to the observed pores. Therefore, although we refrain from classifying NIPAAm as a conventional porogen, our data support its porosity‐inducing effect within the hydrogel system.

Although the Chitosan‐g‐NIPAAm intermediate was not isolated prior to crosslinking, we have provided multiple lines of indirect but converging evidence supporting successful grafting of NIPAAm onto chitosan. The presence of characteristic chemical shifts from both chitosan and NIPAAm in the ^1^H NMR spectrum (Figure 1a) confirms their coexistence in the same polymeric network. FT‐IR spectra (Figure 1b) show a progressive increase in absorbance corresponding to NIPAAm‐specific functional groups (N─H, C═O, and C─N) as its ratio increases, indicating effective incorporation. Furthermore, SEM images (Figure 1c) demonstrate a systematic increase in pore size with higher NIPAAm content, likely due to its hydrophilic character, facilitating water retention and subsequent pore generation upon drying. Together, these complementary spectroscopic and morphological data provide strong evidence that the grafting process was successful within the context and objectives of this study.

Molecular weight analysis was performed using Gel Permeation Chromatography (GPC) on hydrogel samples that had been partially dissolved in dimethyl formamide (DMF). The reported values represent the DMF‐soluble polymer fractions, primarily consisting of unreacted or lightly grafted chitosan‐NIPAAm copolymers, and not the bulk crosslinked network. The results are shown in Table S2a (Supporting Information) derived from chromatogram (Figures S3–S6, Supporting Information). The average molecular weight of CSPP (chitosan/PVA/PVP without NIPAAm) was 52 135 g mol^−1^, while those of the copolymers were 44 061 g mol^−1^ (C2N1), 37 422 g mol^−1^ (C1N1), and 44 099 g mol^−1^ (C1N2). The molecular weight reduction, compared to the initial chitosan (100 000–300 000 g mol^−1^), is likely due to partial chain degradation during the grafting reaction. Although NIPAAm concentration was varied, the molecular weight changes reflect a balance between chain scission and grafting efficiency.

As shown in Table S2b (Supporting Information), the moisture content of CSPP hydrogel was 88.53%. Hydrogels contain a lot of intermolecular and intramolecular hydrogen bonds, so they are rich in water. The moisture content of the chitosan‐g‐NIPAAm hydrogel series was ≈75%. After adding NIPAAm, the hydrogel has a more compact structure, so that the water content of the hydrogel decreases.

**Figure 2:**
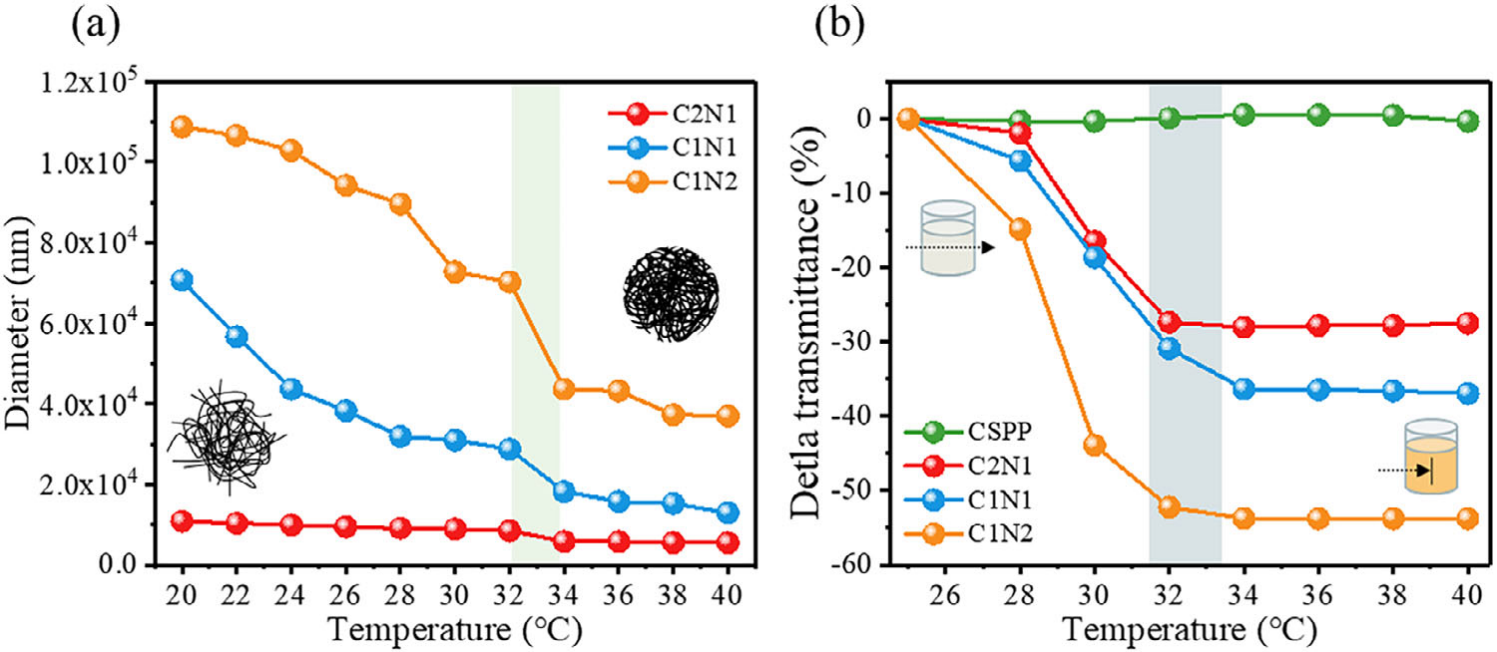
LCST behavior identification of hydrogels. a) The particle size change curve of hydrogel from 20 to 40 °C; b) The change of curve of the light penetration (opacity) of hydrogel from 25 to 40 °C.

LCST is defined as the transition of a molecular chain from an extended coil to a collapsed spherical state in response to temperature changes.^[^ ^40^ ^]^ The change in copolymer particle size with temperature can be studied by dynamic light scattering (DLS). As shown in **Figure** **2**a, below the LCST, the molecular chains of the copolymer are in an extended, linear, hydrophilic state, resulting in a relatively large particle size. When the temperature exceeds the LCST, increased hydrophobic interactions of the isopropyl groups cause the particle size to shrink rapidly, adopting a collapsed conformation.

This smart self‐shrink property is beneficial for wound dressing applications. In the shrunken state, the hydrogel maintains moisture at the wound site even at higher temperatures, preventing disruption of the healing process. Additionally, NIPAAm‐based hydrogels with this smart contraction exert a gentle compressive force on the wound, promoting wound edge approximation and faster closure. Simultaneously, the contracted hydrogel retains moisture effectively, providing a balanced, moist environment essential for cell migration and tissue regeneration. This thermo‐responsive behavior mimics clinical compression therapies used in wound care, offering adaptive conformity and sustained protection while reducing the need for frequent dressing changes and improving patient comfort.

The dependence of the hydrogel on temperature can be observed in Figure 2b. The transmittance of chitosan‐graft‐NIPAAm/PVA/PVP hydrogel decreases with increasing temperature. This result becomes more obvious as the content of NIPAAm increases. The transmittance of CSPP hydrogel without NIPAAm will not be affected by temperature changes. As shown in Table S3 (Supporting Information), the hydrogel is soluble in water and a clear light yellow liquid at 25 °C. When the temperature is increased to 40 °C, the hydrogel becomes opaque and creamy white. Therefore, it is expected that this smart physical color change will be an obvious indicator of body temperature when applied as a wound dressing.

**Figure 3:**
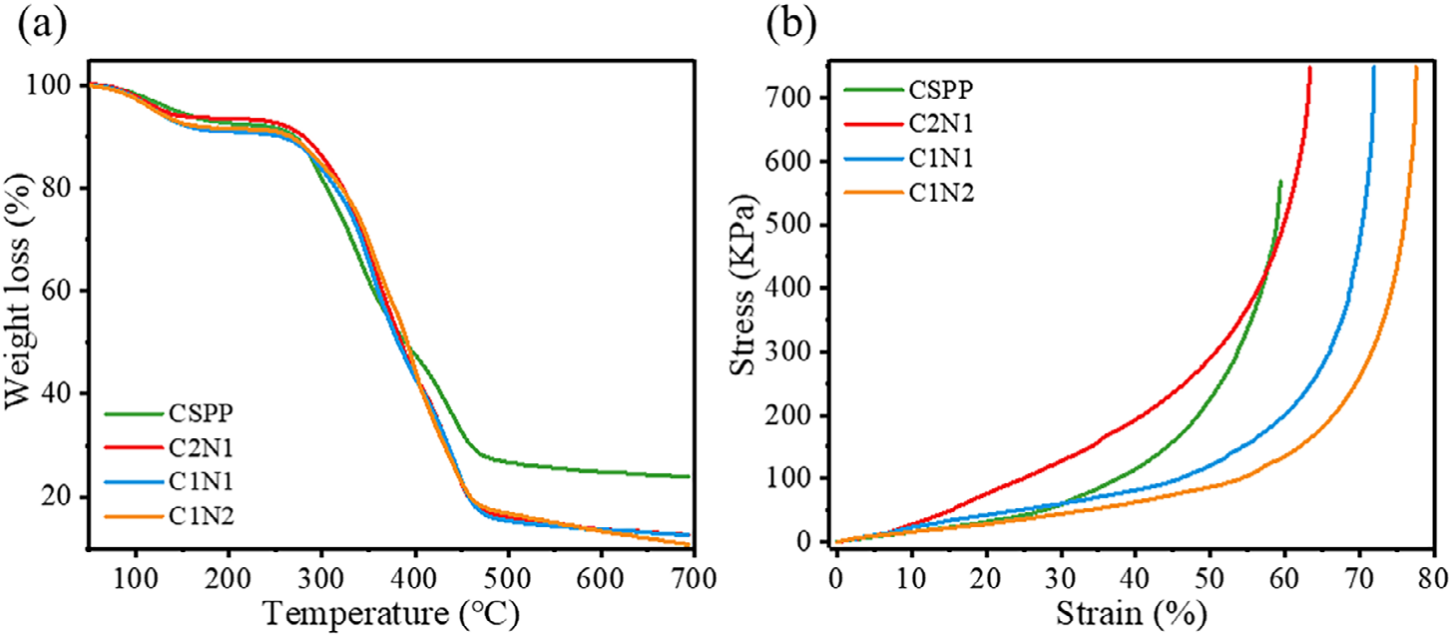
a) TGA curve of hydrogel; b) Stress–strain curve of hydrogel.

The thermal stability of the hydrogel was evaluated by TGA. As shown in **Figure** **3**a, when the temperature raised from 50 to 700 °C, there are three significant weight loss stages. At the first stage, the residual water loss in the hydrogel is between 50 and 200 °C.^[^ ^35^ ^]^ The temperature in the second stage is ≈300 °C, and this stage is the cleavage reaction of the chitosan chain.^[^ ^41^ ^]^ The third stage is close to 439 °C, which is the degradation temperature of residual organic polymer fragments.^[^ ^27^ ^]^

Understanding these thermal degradation stages is essential for assessing the hydrogel's stability during sterilization and storage, ensuring its reliability as a wound dressing material. The melting point and glass transition temperature of the hydrogel are measured by differential scanning calorimetry and dynamic mechanical analysis. The data each are shown in Figure 3a,b. As shown in Table S2c (Supporting Information), a broad endothermic peak is generated at 118–143 °C, which was presumably caused by intermolecular and intramolecular hydrogen bonds. CSPP hydrogel contains only chitosan, semi‐crystalline polymer PVA, and non‐crystalline polymer PVP, and its melting point temperature was 143.17 °C. As the content of NIPAAm increases, the melting point temperature appears at a lower temperature. The melting points of C2N1, C1N1, and C1N2 hydrogels were 139.12, 129.33, and 118.94 °C. The glass transition temperature indicates the temperature at which a material absorbs energy and softens. As shown in Table S2d (Supporting Information), as the content of NIPAAm increases, the glass transition temperature (Tg) needs to appear at a higher temperature. The Tg of CSPP, C2N1, C1N1, and C1N2 hydrogels were 53.63, 57.75, 67.30, and 71.41 °C, respectively. Tg is identified by the peak of the damping factor (Tan δ), calculated as the ratio of the loss modulus (G″) to the storage modulus (G′). Figure S7 (Supporting Information) shows that, when heated from 0 to 100 °C, the loss modulus of the C2N1, C1N1, and C1N2 hydrogels ranges from 0.08 to 1.04 MPa, while the storage modulus ranges from 1.71 to 12.06 kPa. Throughout the temperature range, G′ remains higher than G′′, indicating that the hydrogels maintain dominant elastic behavior. Compared to CSPP hydrogels, the NIPAAm‐containing samples exhibit enhanced viscoelasticity and elasticity, suggesting that NIPAAm contributes significantly to the increase in both G′ and G′′.

G′ is known to be sensitive to both crosslinking density and environmental temperature, especially in thermoresponsive systems containing PNIPAAm. As the temperature approaches or exceeds PNIPAAm's lower critical solution temperature (LCST, ≈32 °C), the polymer network undergoes a volume phase transition that results in increased stiffness and a marked rise in G′ due to enhanced hydrophobic interactions and polymer chain collapse.^[^ ^42^, ^43^ ^]^ In our hydrogel system, thermosensitive behavior is still observed, although the transition does not occur exactly at 32 °C. This deviation is likely due to the presence of other polymers such as chitosan, PVA, and PVP, which influence the network structure and hydration characteristics, thereby shifting the transition temperature.

Overall, these temperature‐dependent mechanical responses further support the suitability of PNIPAAm‐containing hydrogels in biomedical contexts, where body and environmental temperature can naturally modulate material behavior. Overall, these thermal properties provide critical insights into the hydrogel's mechanical performance and durability, which are important for maintaining dressing integrity during application and ensuring a controlled response under physiological conditions.

In this study, compressive mechanical testing was conducted to evaluate the structural integrity of the hydrogels. As shown in the stress–strain curves (Figure 3b) and detailed in Table S2e,f (Supporting Information), the CSPP hydrogel exhibited a compressive strength of 567.94 kPa and a strain at break of 59.37%, which is significantly higher than that of conventional chitosan‐based hydrogels (typically < 200 kPa).^[^ ^44^, ^45^ ^]^ With increasing PNIPAAm content, the mechanical performance further improved: the compressive strength increased from 747.57 kPa in sample C2N1 to 749.31 kPa in C1N2, while the strain at break improved from 63.36% to 77.55%. These enhancements are attributed to the integration of PNIPAAm into the chitosan network, which increases the crosslinking density and structural resilience of the hydrogel.

**Figure 4:**
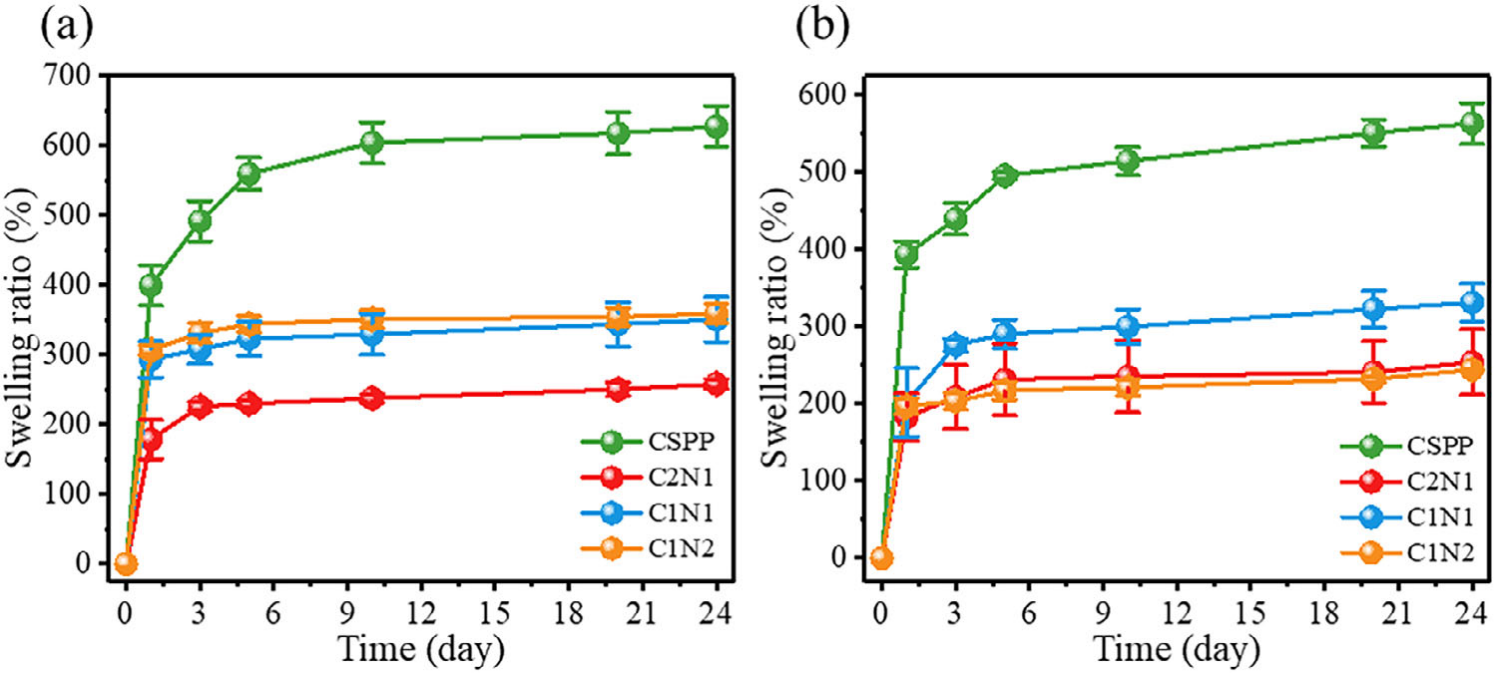
Swelling ratio curve of hydrogels a) at 25 °C and b) at 37 °C. (Mean ± standard error of the mean, *n* = 3).

The most critical feature of hydrogels for wound dressing applications is their ability to absorb large amounts of water, which is primarily governed by the presence of hydrophilic groups in the polymer network. To simulate physiological conditions, we immersed the hydrogels in water at 37 °C. As shown in **Figure** **4**a, the swelling ratio of all hydrogel samples increased over time and reached equilibrium within ≈5 h, which is faster than some hydrogels reported in the literature. For example, a study on PNIPAAm‐DAA‐HA hydrogels showed that the maximum swelling degree was achieved at 36 h.^[^ ^46^ ^]^ After 24 h at 25 °C, the swelling ratios of CSPP, C2N1, C1N1, and C1N2 hydrogels were 627.05 ± 28.97%, 257.92 ± 6.87%, 350.26 ± 32.43%, and 358.86 ± 13.62%, respectively. Under 37 °C conditions (Figure 4b), the swelling ratios slightly decreased to 563.01 ± 26.37%, 253.83 ± 42.25%, 331.11 ± 24.77%, and 244.13 ± 0.71%, respectively.

This temperature‐responsive behavior can be attributed to the thermosensitive nature of PNIPAAm in the hydrogel matrix. At higher temperatures, the increased hydrophobic interactions among isopropyl groups reduce the hydrogel's affinity for water, while disruption of hydrogen bonding further limits water retention. Figure S8 (Supporting Information) shows the temperature‐dependent trend, where swelling ratios of all hydrogel types decreased with increasing temperature: CSPP decreased from 493.30 ± 7.38% to 368.95 ± 10.14%, C2N1 from 266.74 ± 9.98% to 199.50 ± 23.89%, C1N1 from 350.61 ± 7.55% to 240.00 ± 3.32%, and C1N2 from 354.39 ± 5.59% to 240.27 ± 10.90%. This behavior aligns with previous studies showing that PNIPAAm‐based hydrogels exhibit decreased swelling capacity above their LCST due to polymer chain collapse.^[^ ^47^ ^]^

Although the hydrogels exhibit a relatively high swelling ratio, they still maintain temperature‐responsive behavior due to the incorporation of NIPAAm. Upon swelling, the hydrogel matrix absorbs water and reaches an equilibrium state. When the temperature increases beyond the LCST, the hydrophilic‐hydrophobic transition of NIPAAm triggers a network collapse, leading to partial water expulsion and volume reduction even from the swollen condition. This self‐shrinkage does not completely reverse the swelling but modulates the hydrogel's conformation, allowing it to gently conform to the wound and maintain an optimal moist environment. As described in the previous sections, this behavior contributes to wound healing by enhancing tissue regeneration and wound closure.

**Figure 5:**
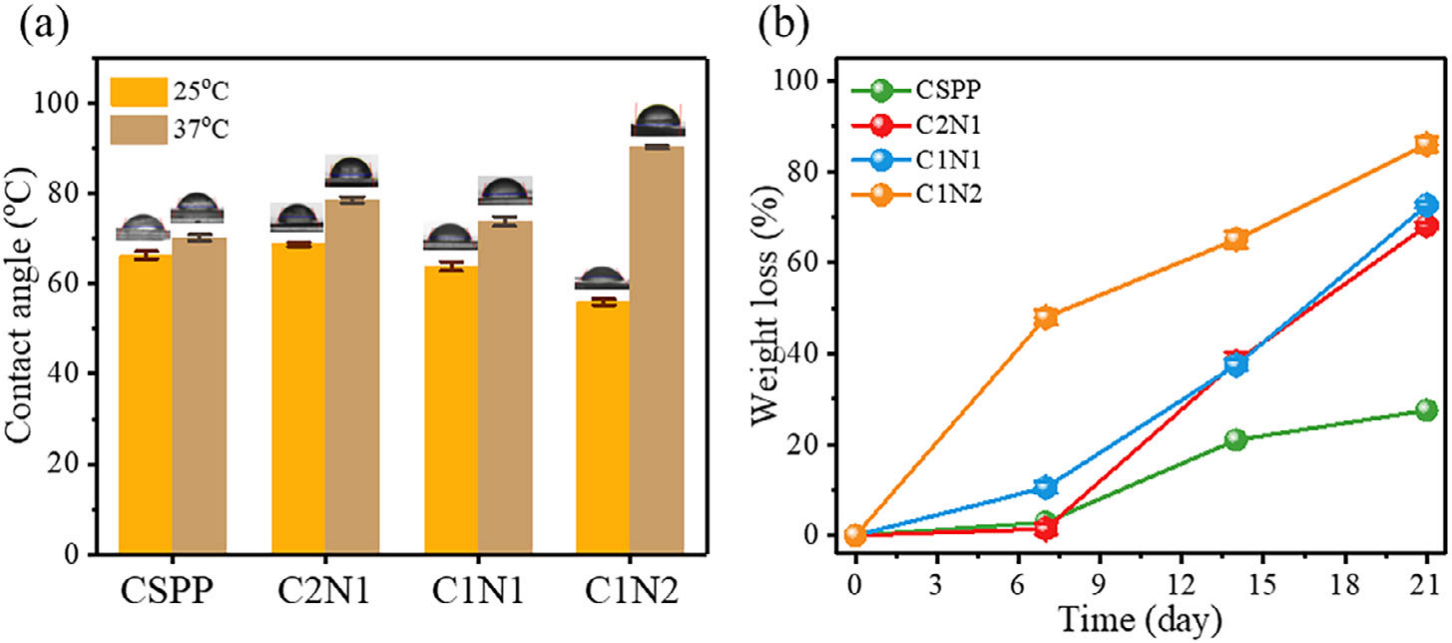
a) The contact angle of the hydrogels at 25 and 37 °C; b) Degradation rate of hydrogels at 7 days, 14 days, and 21 days (mean ± standard error of the mean, *n* = 3).

The hydrophilic and hydrophobic properties of the hydrogel surface were evaluated by measuring the contact angle. As shown in **Figure** **5**a, for the chitosan‐g‐NIPAAm hydrogel series, when the temperature was increased from 25 to 37 °C, the contact angle increased. It was speculated that NIPAAm has an amide group capable of generating hydrogen bonds with water molecules and a hydrophobic isopropyl group. When the temperature was higher than LCST, the molecular cohesion of PNIPAAm was higher than the hydrogen bond force between water molecules, and the hydrophobic force increased. Especially, the C1N2 hydrogel contains more NIPAAm, so the contact angle is increased from 54.99° to 90.12°. This result can correspond to the swelling ratio.

In this study, the hydrogel was immersed in a 37 °C phosphate buffer solution (pH 7.4) solution containing lysozyme. Lysozyme is a common enzyme in organisms that can destroy the cell wall of dissolved bacteria by hydrolysis.^[^ ^48^ ^]^ As shown in Figure 5b, a significant reduction in the weight of the hydrogel can be observed. The in vitro degradation of C1N2 hydrogel achieved a weight loss of 48.01 ± 1.53% on day 7 and a weight loss of 86.02 ± 1.63% on day 21. The result was a gradual increase in weight loss over time to 21 days. It was attributed to the faster hydrolysis rate of the amide group in NIPAAm and PVP in the enzyme solution, so the degradation degree was faster. Chitosan undergoes enzymatic and hydrolytic degradation, yielding low molecular weight oligosaccharides and D‐glucosamine. These degradation fragments are well‐documented to be non‐toxic and are even bioactive, supporting wound healing, antimicrobial activity, and cell proliferation in various biomedical applications.^[^ ^49^ ^]^ PNIPAAm may produce small amide‐containing fragments upon hydrolysis, which have been reported to have low cytotoxicity in dilute concentrations.^[^ ^50^, ^51^ ^]^

In our design, the hydrogel was intentionally formulated with moderate adhesiveness—sufficient to remain securely in place over the wound while avoiding excessive adhesion that could cause tissue damage or pain upon removal. Adhesion was qualitatively evaluated by placing 2 cm × 3 cm hydrogel pieces onto a stainless‐steel plate and human skin (finger), where they adhered without detachment, demonstrating adequate cohesive and adhesive properties for both handling and wound coverage (Figure S9, Supporting Information).

### 2.2 Characterization of AgNPs and AgNPs‐Loaded Chitosan Hydrogels

AgNPs were synthesized using a co‐reduction method with sodium borohydride and trisodium citrate. As shown in Figure S10a (Supporting Information), the AgNP solution exhibited a clear yellow color, and the formation of nanoparticles was confirmed by UV–vis spectroscopy, showing a characteristic absorption peak at 397 nm. The full width at half maximum was 57.11 nm, indicating good particle uniformity. When incorporated into the hydrogel, the absorption peak shifted slightly to 401 nm, while no peak was observed in the hydrogel without AgNPs, confirming successful loading.

Transmission electron microscopy images in Figure S10b (Supporting Information) reveal spherical AgNPs with an average size of ≈10 nm. DLS further confirmed the particle size to be ≈10.95 nm (Figure S10c, Supporting Information). To verify antibacterial activity, the synthesized AgNPs showed a clear inhibition zone against *Escherichia coli* (*E. coli*) and *Staphylococcus aureus* (*S. aureus*) on agar plates (Figure S10d–f, Supporting Information).

The driving force for incorporating AgNPs into the hydrogel matrix lies in the ability of the hydrogel network to act as a carrier and reservoir, enabling sustained and localized release of Ag^+^ ions at the wound site. In this study, AgNPs of ≈10 nm were employed based on previous reports showing that particles in the 5–15 nm range offer an optimal balance between potent antibacterial efficacy and low cytotoxicity. Smaller AgNPs (≈10 nm) provide a higher surface area‐to‐volume ratio, facilitating enhanced interaction with bacterial membranes and more efficient Ag^+^ release at lower concentrations.^[^ ^52^, ^53^ ^]^ Moreover, they diffuse more uniformly within the hydrogel matrix and wound tissue, while their small size reduces aggregation and ensures stable loading in hydrophilic networks.^[^ ^54^ ^]^ Furthermore, the hydrogel's porous, hydrated structure facilitates uniform dispersion and physical entrapment of AgNPs without aggregation, which is critical for maintaining consistent antibacterial performance. In some formulations, functional groups in the hydrogel (e.g., hydroxyl, carboxyl, or amide groups) may also interact via coordination or hydrogen bonding with AgNPs, providing additional stabilization within the matrix.^[^ ^55^, ^56^ ^]^ This controlled incorporation prevents burst release, reduces cytotoxicity associated with free nanoparticles, and ensures prolonged antimicrobial protection, which is a critical requirement for wound healing applications.^[^ ^29^, ^57^ ^]^

To evaluate Ag^+^ release, all samples were immersed in water, and the AgNPs’ corresponding peak (Figure S10a, Supporting Information) was monitored over time. After 24 h, the peak intensity decreased from the initial 100% to 98.0%, 95.9%, 82.4%, and 80.2% for CSPP/AgNPs, C2N1/AgNPs, C1N1/AgNPs, and C1N2/AgNPs, respectively (Figure S11, Supporting Information). The greater NIPAAm content in the hydrogel likely increases the contact surface between AgNPs and water, thereby facilitating release from the hydrogel matrix, which is consistent with the SEM image in Figure 1c. In contrast, AgNPs loaded on filter paper exhibited poor release control, losing 76.2% within the first 24 h, confirming that the hydrogel matrix can effectively trap AgNPs and regulate their release. After 54 h, all hydrogels were swollen; however, the AgNPs’ peak intensities remained at 82.9%, 73.3%, 71.6%, and 69.2% for CSPP/AgNPs, C2N1/AgNPs, C1N1/AgNPs, and C1N2/AgNPs, respectively, indicating that swelling may affect the release rate but still prevents burst release.

**Figure 6:**
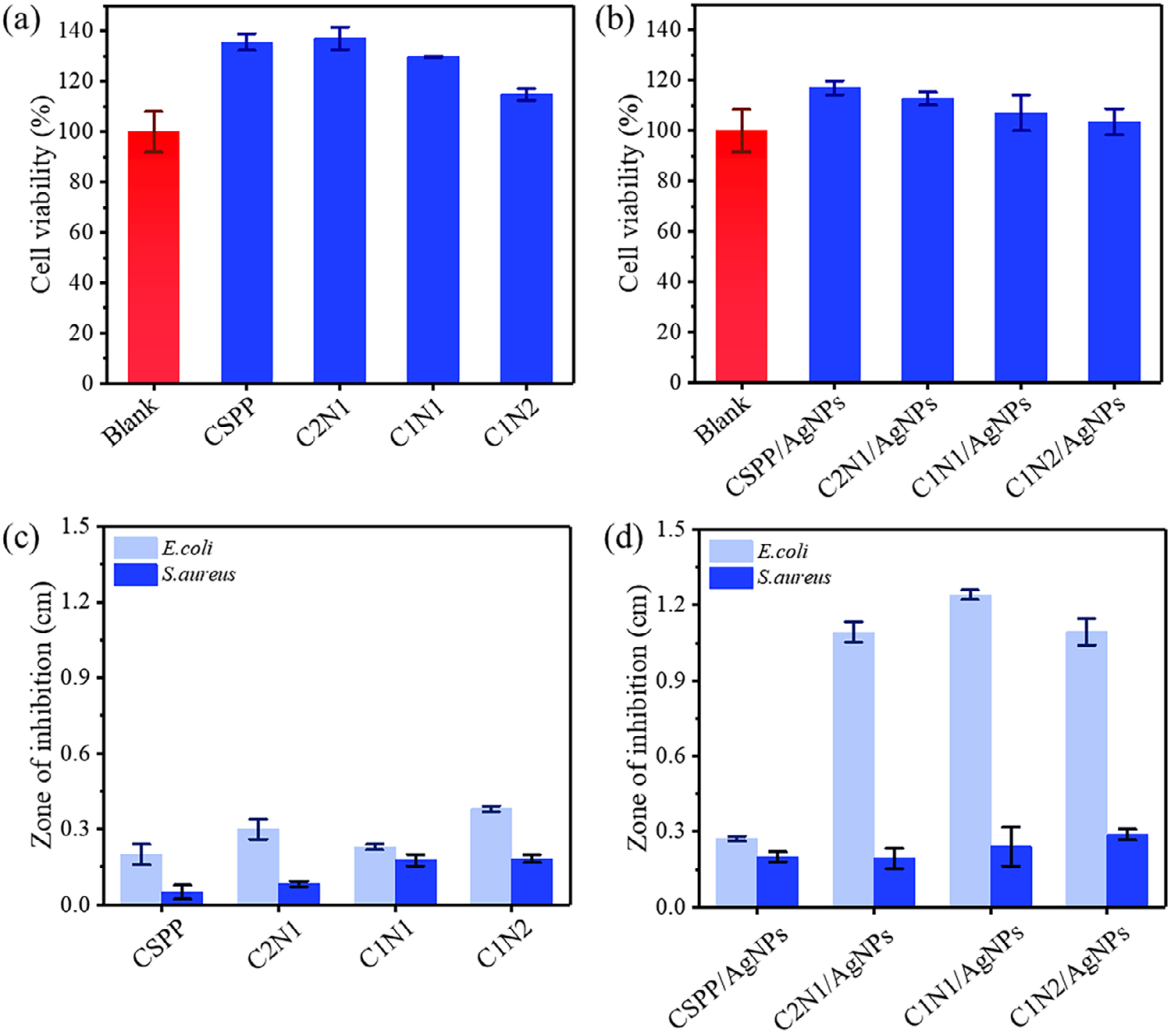
a,b) L929 cell viability of hydrogels (CSPP, C2N1, C1N1, C1N2) with/without AgNPs and blank control, measured by MTT assay (mean ± SEM, *n* = 3). c,d) Inhibition zones of the same hydrogels against *E. coli* and *S. aureus* (mean ± SEM, *n* = 3).

The cytocompatibility of AgNPs‐loaded chitosan hydrogels was assessed by direct contact tests using the MTT assay. As shown in **Figure** **6**a,b, all hydrogels demonstrated high metabolic activity (> 90%) after 72 h of incubation, indicating good biocompatibility for wound dressing applications. It is worth noting that in direct contact MTT assays, the measured absorbance reflects not only cell viability but also cell adhesion and proliferation, since only metabolically active, adherent cells contribute to the formazan signal. Slight reductions in viability were observed with increasing NIPAAm content, but remained within acceptable limits. This minor decline may be attributed to the higher concentration of PNIPAAm, which can affect the local microenvironment through increased hydrophobic interactions and lower hydration at physiological temperature. These changes may reduce nutrient diffusion or cell–substrate interactions, slightly impacting cell viability.^[^ ^42^ ^]^ Furthermore, unreacted monomers or oligomers at higher NIPAAm ratios may contribute to mild cytotoxic effects if not completely removed during purification.^[^ ^50^, ^51^ ^]^ Among all samples with AgNPs (Figure 6b), a slight reduction in cell metabolic activity was observed compared to samples without AgNPs (Figure 6a), but the values remained within a safe range. In our formulation, AgNPs were incorporated at low concentrations and confined within the hydrogel network, minimizing the rate and extent of Ag^+^ ion release. Literature reports confirm that such embedded AgNPs show significantly reduced cytotoxic and inflammatory potential compared to free nanoparticles.^[^ ^58^ ^]^

To evaluate antibacterial activity, the hydrogels were tested against *E. coli* and *S. aureus*. As shown in Figures 6c and S12a (Supporting Information), all hydrogel without AgNPs show a slight antibacterial effect regarding the chitosan polymer ability to hinder bacterial.^[^ ^59^ ^]^ On the other hand, AgNPs‐loaded hydrogels Figures 6d and S12b (Supporting Information) exhibited significant antibacterial activity against *E. coli*, with larger inhibition zones compared to CSPP hydrogel due to the higher loading and release capacity of the chitosan‐g‐NIPAAm network. The antibacterial activity of AgNP‐loaded hydrogels (Figure 6d) reflects the combined effects of AgNPs alone (Figure S10d–f, Supporting Information) and the chitosan‐based hydrogel (Figure 6c).

However, the inhibition effect against *S. aureus* was comparatively weaker. This reduced efficacy may be attributed to the thicker peptidoglycan layer in the *S. aureus* cell wall, which can hinder the diffusion and interaction of Ag^+^ ions with the bacterial membrane.^[^ ^30^ ^]^ Additionally, the release profile of Ag^+^ from the hydrogel matrix may not have reached the concentration required for effective action against Gram‐positive bacteria. Despite this, the observed antibacterial effect still suggests potential, especially when combined with the hydrogel's other beneficial properties for wound healing.

In our system, AgNPs were incorporated into the polymer suspension via simple physical mixing. Antibacterial testing showed that the CSPP hydrogel (without chitosan) exhibited lower antibacterial activity than the chitosan‐containing formulations (C2N1, C1N1, and C1N2). As shown in the SEM images and Ag^+^ release profile (Figures 1c; S11, Supporting Information), CSPP displayed a less porous structure, whereas the chitosan‐containing hydrogels exhibited increased porosity. Since porosity influences both water diffusion and nanoparticle mobility, we infer that the presence of chitosan reduces AgNP retention and increases the likelihood of Ag^+^ release from the polymer matrix.

### 2.3 Wound Healing Experiments

**Figure 7:**
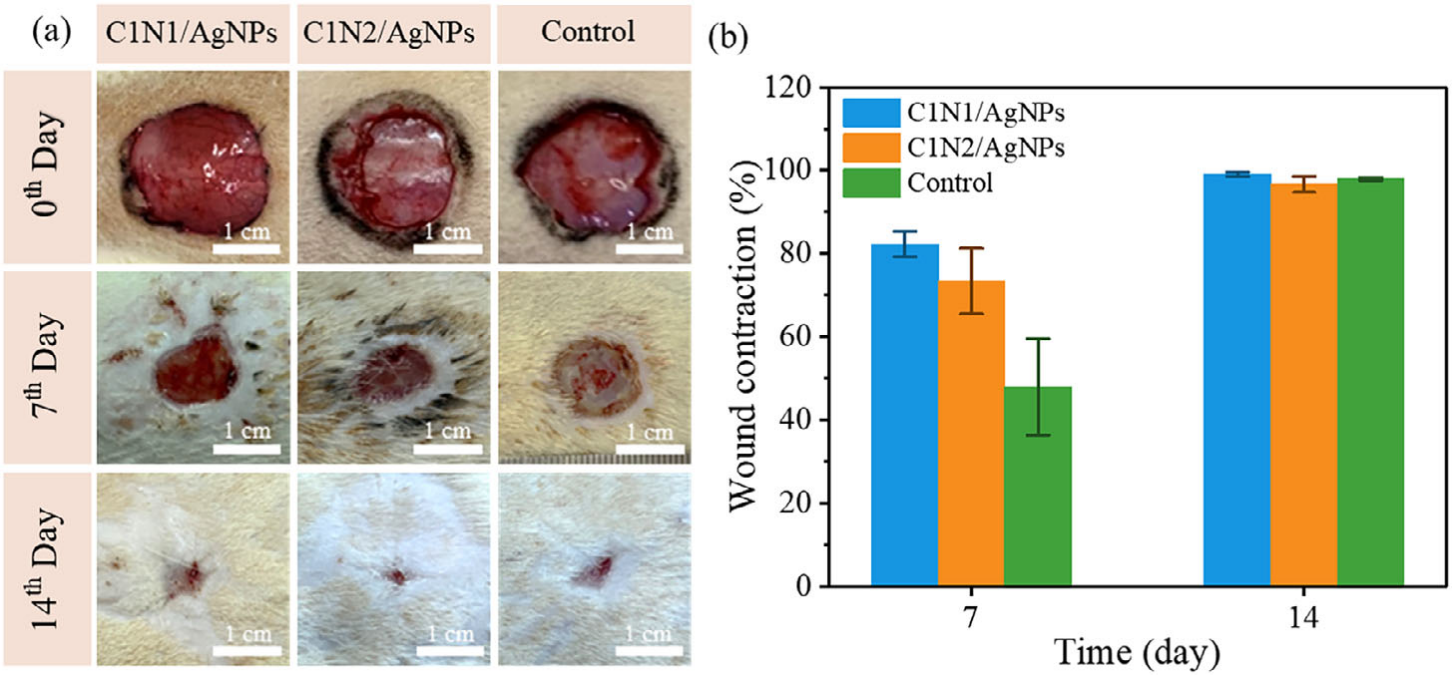
a) Photographs of the wound healing process treated with different wound dressings at 0, 7, and 14 days post excision injury; b) Wound contraction of use C1N1/AgNPs, C1N2/AgNPs hydrogels, and control (mean ± standard error of the mean, *n* = 5 wounds per group). Wound area was measured using ImageJ, with details provided in the Supporting Information.

Based on the above experiments, it is known that C1N1 and C1N2 hydrogels have excellent results in basic tests. Therefore, the skin tissue repair performance of hydrogels was studied using an Sprague‐Dawley (SD) rat with back skin full‐thickness defection. **Figure** **7**a shows the macroscopic view of the wounds from the three treatment groups on days 0, 7, and 14. C1N1/AgNPs hydrogel had a faster wound contraction rate than that of C1N2/AgNPs hydrogel and control. In vivo experiments on a rat full thickness wound model showed that the hydrogel conformed to the wound bed and remained attached to the wounded tissue. While it performs better with auxiliary support (fixed with a hydrocolloid thin dressing and transparent film dressing frame style), it still has the potential to be attached (Figure S13, Supporting Information).

On the 7th day, the wound healing rate of C1N1/AgNPs, C1N2/AgNPs hydrogel, and the control group was 82.33 ± 3.06%, 73.43 ± 7.87%, and 47.97 ± 11.6% in Figure 7b. On the 14th day, all the wounds in the three groups were almost closed, and there was no significant difference among them with the healing rate of C1N1/AgNPs, C1N2/AgNPs hydrogel, and the control group was 99.11 ± 0.50%, 96.69 ± 1.89%, and 97.97 ± 0.34%. It can be proved that the Ag^+^ ions released in the C1N1/AgNPs hydrogel achieve antibacterial effects and do not cause bacterial infection of the wound. The LCST promotes wound closure in SD rats to obtain a higher healing effect.

To quantitatively evaluate the wound healing performance of the hydrogels, a two‐way ANOVA was conducted to assess the effects of hydrogel type (C1N1/AgNPs, C1N2/AgNPs, and control) and treatment duration (Day 7 vs Day 14) on wound closure rates (Table S4a, Supporting Information). The analysis revealed a significant effect of sample type (*F* = 4.70, *p* = 0.020) and a highly significant effect of treatment time (*F* = 38.65, *p* = 2.00 × 10^−6^), indicating that both hydrogel composition and healing time significantly influenced wound recovery. The interaction between sample type and time point also yielded a significant result (*p* = 0.02), suggesting that the impact of the hydrogel formulation varies across different time points.

To further identify the specific differences among groups, a Tukey post hoc test was performed (Table S4b, Supporting Information). The results demonstrated that the control group at Day 7 exhibited significantly lower wound closure compared to the C1N1/AgNPs Day 7, C1N1/AgNPs Day 14, and C1N2/AgNPs Day 14 groups. A significant difference was also observed between control Day 14 and control Day 7, confirming the natural progression of healing over time. Interestingly, no significant differences were observed between Day 7 and Day 14 within the C1N1/AgNPs and C1N2/AgNPs groups, suggesting that the majority of wound closure occurred within the first 7 days post‐treatment. This early‐phase acceleration of healing likely reflects the bioactivity and favorable physicochemical properties of the hydrogels, which may promote rapid tissue regeneration and re‐epithelialization. These findings indicate that the hydrogel formulations, particularly C1N1/AgNPs and C1N2/AgNPs, enhance early‐stage wound healing compared to the untreated control, and that their effect plateaus beyond the initial week, underscoring their potential as efficient short‐term wound dressings.

**Figure 8:**
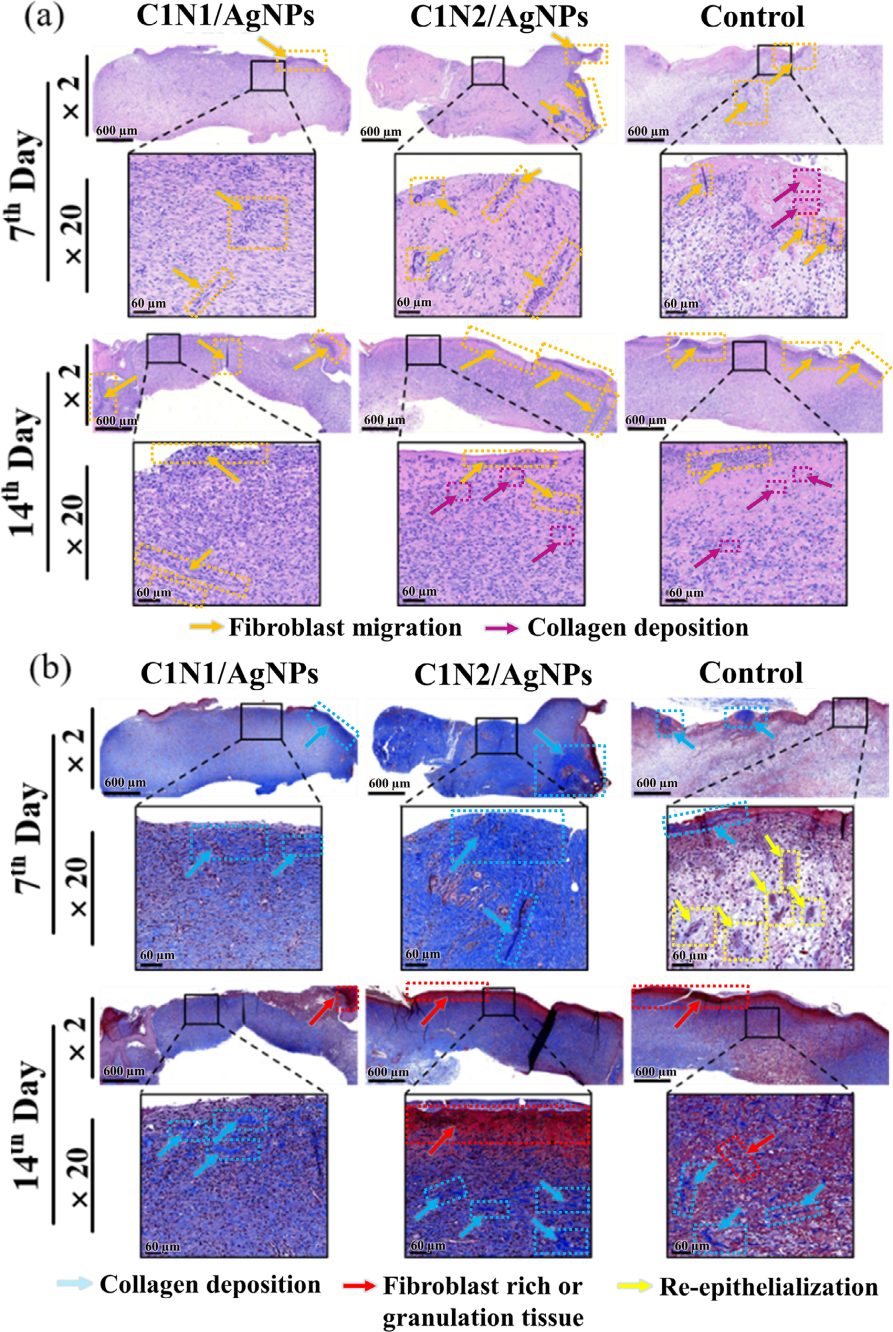
Tissue section analysis of C1N1/AgNPs, C1N2/AgNPs hydrogels, and the control group on the 7th and 14th day. a) The images of H&E staining. b) Representative photographs of MT staining of healed wounds for C1N1/AgNPs, C1N2/AgNPs, and the control group on 7th and 14th day, scale bar = 600 µm. (The enlarged images in the box area; scale bar = 60 µm).

To evaluate the healing effect of the dressings from a histological perspective, hematoxylin and eosin (H&E) and Masson's Trichrome (MT) staining were performed on regenerated skin tissues collected on the 7th and 14th days. The magnified images allowed detailed observation of tissue organization, helping to assess the extent of wound healing. In the H&E‐stained sections (**Figure** **8**a), granulation tissue—composed of fibroblasts and growth factors—was clearly observed, serving as a key indicator of tissue regeneration. This tissue appeared as loosely organized, cell‐rich regions beneath the scab or regenerating epithelium, particularly prominent on Day 7 (orange arrows). Fibroblasts were identified by their elongated, spindle‐shaped nuclei stained purple (purple arrows), indicating active migration and proliferation. Varying degrees of inflammatory cell infiltration, fibroblast activity, and granulation tissue formation were noted among the groups, suggesting that the treated groups facilitated a more accelerated healing response in the early phase of repair.^[^ ^15^, ^60^ ^]^

By day 14, H&E staining revealed the presence of numerous new capillaries and uniformly organized fibrous tissue within the wound bed, indicating active tissue remodeling.^[^ ^61^ ^]^ These findings are consistent with the wound contraction results presented in Figure 7. However, due to the advanced healing state at this time point, histological differences between groups became less distinguishable. Interestingly, collagen‐like structures appeared more prominent in the control group at Day 14, possibly reflecting a delayed transition into the remodeling phase. In contrast, the hydrogel‐treated groups had already progressed through granulation and entered the remodeling phase earlier, resulting in more organized tissue architecture.

In the MT‐stained sections (Figure 8b), collagen deposition and structural remodeling were assessed. This staining method highlights collagen fibers and muscle tissue by differentiating them in color. All groups exhibited increased collagen content from day 7 to day 14,^[^ ^15^ ^]^ but the C1N1/AgNPs and C1N2/AgNPs hydrogel groups displayed stronger, blue‐stained collagen fibers (light blue arrows), indicating more mature extracellular matrix (ECM) regeneration. In the control group on Day 7, purple nuclear bundles (indicated by yellow arrows) were observed, suggesting active cellular proliferation related to re‐epithelialization or inflammatory activity. Additionally, the presence of a continuous, red‐stained outer epidermal layer—particularly evident in the C1N1/AgNPs group—indicated improved re‐epithelialization and more advanced wound closure.

**Figure 9:**
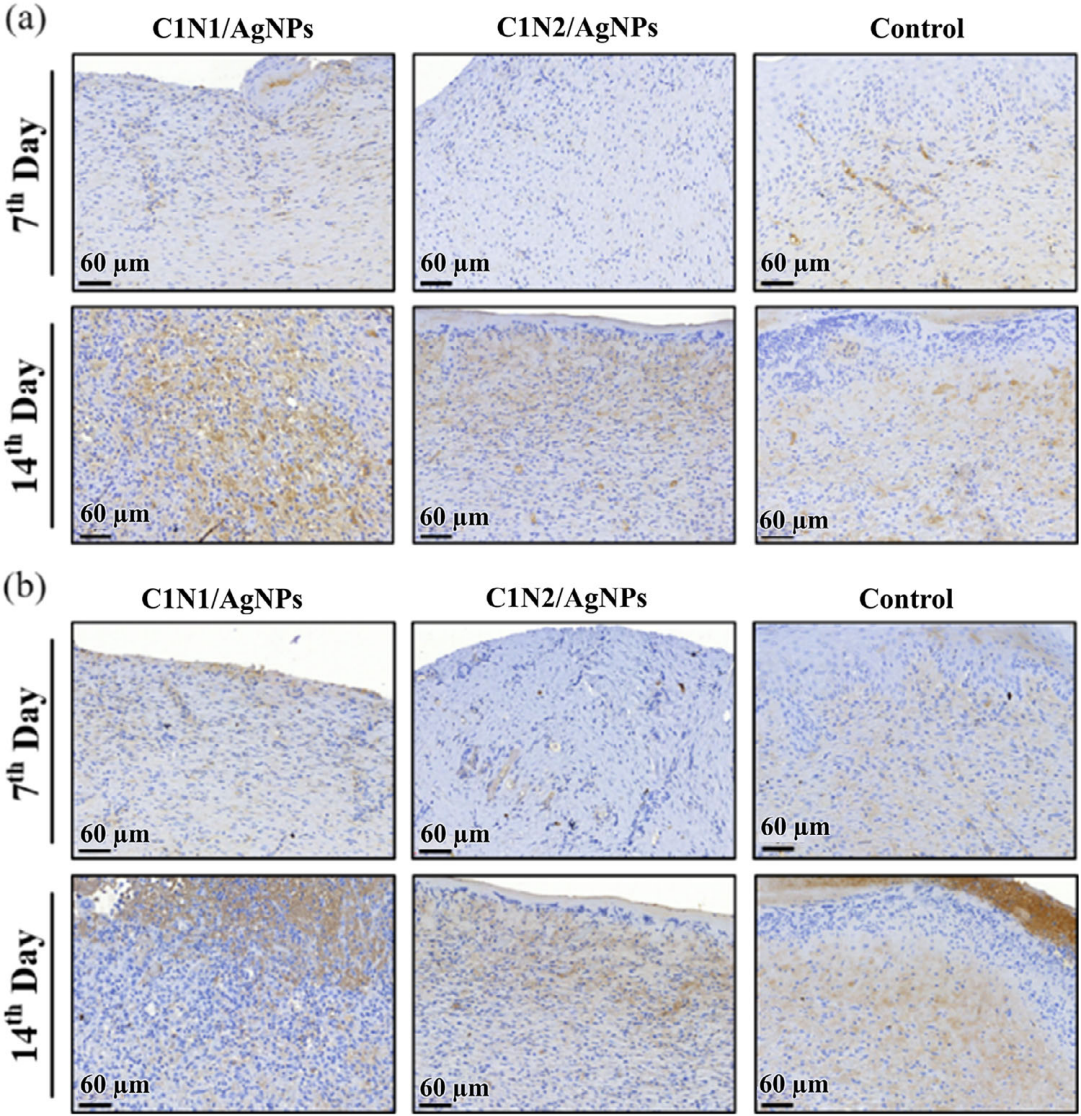
Immunohistochemical staining of wound tissue sections from C1N1/AgNPs, C1N2/AgNPs hydrogel‐treated, and control groups on the 7th and 14th days. a) CD31 staining (brown), a marker for endothelial cells, shows increased expression in the C1N1 group by day 14, indicating enhanced angiogenesis. b) CD68 staining (brown), used to identify macrophages, reveals a greater presence in the C1N1/AgNPs group on day 7, with further accumulation by day 14, suggesting a more active inflammatory and tissue remodeling response compared to the other groups. Scale bar = 60 µm. Positive expression is indicated by brown chromogenic staining.

The immunohistochemical staining of CD31 and CD68 was carried out to detect the marker for endothelial cells and macrophages on the 7th and 14th day. In **Figure** **9**a, Platelet endothelial cell adhesion molecule (CD31) can prove the presence of endothelial cells in tissue sections and assess the degree of angiogenesis. On the 7th day, the content of CD31 in wound tissues was lower in the three groups, but by the 14th day, a significant amount of CD31 was observed from the C1N1/AgNPs hydrogel. As shown in Figure 9b, after CD68 immunostaining, macrophages were found in the C1N1/AgNPs hydrogel on the 7th day, but the C1N2/AgNPs hydrogel and the control group were almost not observed. By the 14th day, the macrophage content of the three groups gradually increased, which was the most for C1N1/AgNPs hydrogel.^[^ ^62^ ^]^ These findings support the wound contraction data presented in Figure 7. Specifically, the enhanced CD31 expression on day 14 in the C1N1/AgNPs group indicates improved angiogenesis, which correlates with the faster wound closure observed macroscopically. Similarly, the early infiltration of CD68‐positive macrophages in the C1N1/AgNPs‐treated wounds suggests a more robust initial immune response, promoting faster tissue remodeling and healing.

Interestingly, in the control group at day 14, CD68 staining appeared particularly intensely near the surface region, which may suggest a localized accumulation of macrophages and a discrete site of inflammation. This phenomenon has been observed in other wound healing studies, where macrophages tended to cluster at the wound surface during prolonged or unresolved inflammatory phases.^[^ ^63^, ^64^, ^65^ ^]^ This could indicate that the untreated wound surface elicited a more pronounced immune response, possibly due to prolonged exposure to external irritants or lack of protective coverage, leading to sustained macrophage activation and delayed transition to the proliferative phase.^[^ ^66^, ^67^ ^]^

The accelerated wound healing and enhanced tissue regeneration observed with C1N1/AgNPs and C1N2/AgNPs hydrogels compare favorably with previously reported hydrogel dressings. For example, Ag^+^‐ion loaded hydrogels have demonstrated effective antibacterial properties and enhanced wound healing, but often face challenges with cytotoxicity at high silver concentrations.^[^ ^68^ ^]^ Our C1N1/AgNPs hydrogel achieves a balanced Ag^+^ release profile that maintains antimicrobial efficacy without harming surrounding tissue. Thermo‐responsive hydrogels exhibiting an LCST are an emerging class in wound care, where temperature‐triggered physical changes can enhance wound contraction and closure.^[^ ^69^ ^]^ The LCST‐induced wound contraction demonstrated by our C1N1/AgNPs hydrogel provides an active healing mechanism that outperforms traditional passive dressings. Additionally, the immunohistochemical results indicate enhanced angiogenesis and macrophage modulation in C1N1/AgNPs‐treated wounds, aligning with findings that controlled inflammatory responses and neovascularization are essential for quality tissue regeneration.^[^ ^70^, ^71^ ^]^ This multifaceted effect underscores the potential of our hydrogel system as a next generation wound dressing.

## 3 Conclusion

Through simple synthesis, the thermosensitive hydrogel of chitosan‐g‐NIPAAm crosslinked PVA/PVP blend polymer was successfully produced and loaded with AgNPs to improve antibacterial activity. After a series of tests, the hydrogel was proven to have a network of pores with a high swelling rate in water. It has biodegradability and good biocompatibility. Among them, C1N1/AgNPs hydrogel has great potential to be used as an antibacterial wound dressing material. Finally, this work findings exhibit a powerful hydrogel formulation for chitosan hydrogel applied as high performance wound dressing.

## Conflict of Interest

The authors declare no conflict of interest.

## Supporting information



Supporting Information

## Data Availability

The data that support the findings of this study are available from the corresponding author upon reasonable request.
