# Supplementary material for: Formulating Smart All‐in‐One Chitosan Hydrogel for High Performance Wound Dressing
Source: Adv Healthc Mater. 2025 Oct 6;15(4):e02971. doi: 10.1002/adhm.202502971 (PMC12836463; doi:10.1002/adhm.202502971)
Supplement: Supplementary file 1 — Supporting Information [file ADHM-15-0-s001.docx]

Supporting Information

Formulating Smart All-in-One Chitosan Hydrogel for High Performance Wound Dressing

Chia-Chi Lin^+^, Magattang Gafur Muchlis^+^, Ren-Jei Chung, Ssu Yu Huang, Michal Martinka, Syang-Peng Rwei, Aivaras Kareiva, Jen-Chang Yang, and Chun Che Lin*

C.-C Lin, A. M. G. Muchlis, S.-P. Rwei, C. C. Lin

Institute of Organic and Polymeric Materials, National Taipei University of Technology, Taipei 106, Taiwan.
E-mail: cclin0530@mail.ntut.edu.tw

R.-J. Chung

Department of Chemical Engineering and Biotechnology, National Taipei University of Technology, Taipei 10608, Taiwan.

S. Y. Huang

Industrial Technology Research Institute, Hsinchu City 300044, Taiwan.

M. Martinka

Department of Clothing Technology, Technical University of Liberec, Liberec 1 461 17 Liberec, Czech Republic.

C.-C. Lin, S.-P. Rwei, C. C. Lin
Research and Development Center for Smart Textile Technology, National Taipei University of Technology, Taipei 106, Taiwan.

A. Kareiva

Institute of Chemistry, Vilnius University, Vilnius 03225, Lithuania.

J.-C. Yang

Graduate Institute of Nanomedicine and Medical Engineering, Taipei Medical University, New Taipei City 235, Taiwan.

Experimental Methods

**Materials and Reagents**

Chitosan (*M*_n_ = 100,000−300,000 g/mol, deacetylated ≥ 75%), sodium hydroxide (NaOH, purity ≥ 98%) and citric acid trisodium salt (TSC, purity ≥ 98%) were obtained from Sigma-Aldrich. N-Isopropylacrylamide (NIPAAm, purity 99%), polyvinylpyrrolidone (PVP, *M*_w_ = 50,000 g/mol) and N,N,N',N'-tetramethyl-ethane-1,2-diamine (TEMED, purity 99%) were obtained from Acros Organics. Polyvinyl alcohol (PVA, *M*_w_ = 88,000−97,000 g/mol, purity 98−99%) was obtained from Alfa Aesar. Silver nitrate (AgNO_3_, purity 99.8%) and Sodium borohydride (NaBH_4_, purity 98%) were obtained from Honeywell Flika. Acetic acid (purity 99%) was obtained from Scharlau. Ammonium persulphate (APS, purity ≥ 98%) was obtained from J.T.Baker.

**Preparation of Chitosan-g-NIPAAm/PVA/PVP Hydrogel**

A 2% (w/v) chitosan suspension was obtained and dissolved in a 1% (v/v) acetic acid solution while stirring at room temperature for 12 hours. After that, NIPAAm (0.25, 0.5, and 1.0 g) was added under nitrogen atmosphere then added with 0.131 mmol APS and 0.2 mmol TEMED and stirred well.

A 10% (w/v) PVA/PVP aqueous solution was prepared and heated to 90 °C until PVA and PVP were fully dissolved. The PVA/PVP solution was poured into the chitosan solution. The weight ratio of chitosan solution and PVA/PVP solution were 1:5, then heated at 60 °C for 2 hours, and kept stirring at room temperature for 12 hours. The resulting solution was poured into a petri dish and put in a 50 °C oven to remove the solvent. It was soaked in 12% NaOH solution for 1 hour to form a hydrogel. The hydrogel then was rinsed with deionized water until the pH was neutral and stored the hydrogel in a phosphate buffer solution (PBS). In this study, chitosan:NIPAAm ratio in grafted Chitosan-g-NIPAAm copolymer was regulated as 2:1, 1:1, and 1:2 named as C2N1, C1N1, and C1N2. On the other hand, copolymer without NIPAAm grafting (Chitosan/PVA/PVP) was named as CSPP in this paper.

**Synthesis of AgNPs Loaded Chitosan-g-NIPAAm/PVA/PVP Hydrogels**

The AgNPs in this study were synthesized according to the method reported in the literature.^[1]^ NaBH_4_ was used as the primary reducing agent and TSC as the secondary reducing agent and stabilizer to synthesize AgNPs. The reduction process was completed at 60 °C and 90 °C, respectively. NaBH_4_ (2×10^-2^ M, 24 mL) and TSC (2×10^-2^ M, 24 mL) aqueous solution were mixed in the dark and heated to 60 °C for 30 minutes while stirring vigorously to ensure the uniformity of the solution. Then AgNO_3_ solution (1×10^-2^ M, 2 mL) was added drop by drop and the temperature was raised to 90°C. When the temperature reached 90 °C, the pH value of the solution was adjusted to 10.5 with 0.1 M NaOH, and the solution was heated continuously for 20 minutes. The nanoparticle suspension was cooled at room temperature. AgNPs suspension was centrifuged at 15,000 rpm for 30 minutes to remove the unreacted reductants. Finally, the obtained AgNPs were stored in a refrigerator at 4 °C until use. 10 mL of AgNPs was added to the hydrogel and stirred to distribute the AgNPs in the hydrogel evenly, then celled as CSPP/AgNPs, C2N1/AgNPs, C1N1/AgNPs, and C1N2/AgNPs.

**Characterization of AgNPs**

The absorption position of AgNPs was detected on an ultraviolet-visible absorption spectrometer (UV-Vis, Jasco V-730) (range 300-700 nm). A transmission electron microscope (TEM, Hitachi HT-7700) was used to analyze the morphological characteristics and size of silver nanoparticles. Dynamic Light Scattering (DLS, Brookhaven 90Plus PALS), when the 640 nm laser light is radiated on the silver nanoparticles, the deviation is generated, and the average particle size is recorded and calculated by the detector.

**Characterization of Chitosan-g-NIPAAm Hydrogels**

The grafting of NIPAAm onto chitosan was carried out *in situ*, and the resulting Chitosan-g-NIPAAm copolymer was directly used in the hydrogel formulation without isolating the intermediate grafted product. Although isolation could allow further characterization, it was not pursued due to purification complexity, as our focus was on developing a functional hydrogel for wound healing.

The chemical structure of the hydrogel was confirmed using Proton Nuclear Magnetic Resonance (^1^H NMR, Bruker AV 500 MHz) in D_2_O. FT-IR spectroscopy (Perkin Elmer Spectrum Two) was used to identify characteristic functional groups in the dry hydrogels. Molecular weight was determined via Gel Permeation Chromatography (GPC, Viscotek GPCmax VE 2001) using DMF as the solvent.

Morphological analysis was performed using Scanning Electron Microscopy (SEM, Hitachi TM4000Plus). Dried samples were mounted on conductive carbon tape, fixed on an SEM stub, and sputter-coated with a thin layer of gold.

Thermal stability was analyzed using Thermogravimetric Analysis (TGA, Netzsch TG209 F3) by heating from room temperature to 700 °C at a rate of 10 °C/min under an argon atmosphere. Differential Scanning Calorimetry (DSC, Perkin Elmer DSC 8000) was conducted from 50 °C to 200 °C at a heating rate of 10 °C/min under a nitrogen atmosphere.

The mechanical properties of the dried hydrogels were evaluated using a Dynamic Mechanical Analyzer (DMA, TechMax EXSTAR 6000) in compression mode. Measurements were performed at a heating rate of 5 °C min⁻¹ from –20 °C to 120 °C. Stress-strain analysis was conducted by applying a compressive force at a rate of 1000 mN min⁻^1^ from 0 to 9000 mN at 25 °C. Surface wettability was evaluated using a contact angle analyzer (Phoenix 300) to determine hydrophilicity and hydrophobicity.

**Moisture Content Measurements**

According to published procedures, the method for measuring the moisture content of the hydrogel has been determined.^[2,3]^ The weight of hydrogel was measured before and after drying in the oven. The water content of hydrogels was calculated according to the following formula (1):

$\text{Moisture content }\left( \text{\%} \right)\text{ }\text{=}\text{ }\frac{\text{W}_{\text{w}} \text{-}\text{ }\text{W}_{\text{d}}}{\text{W}_{\text{w}}}\text{ }\text{×}\text{ }\text{100\%}$ (1)

where W_w_ is the weight of the moist hydrogel and W_d_ is the initial weight of the hydrogel after drying. Three times measurements were performed for each sample and the average value was used.

**Swelling Ratio Test**

According to published procedures, the swelling behavior of hydrogels is determined by equilibrium swelling studies.^[4,5]^ In order to measure the swelling behavior, the pre-weighed hydrogel was immersed in deionized water and taken out at different time intervals. After removing the excess surface water with filter paper, the weight of the swollen sample was measured. The swelling ratio of the hydrogel was defined using the following equation (2):

$\text{Swelling ratio }\left( \text{\%} \right)\text{ }\text{=}\text{ }\frac{\text{W}_{\text{t}}\text{(e)}\text{ }\text{-}{\text{ }\text{W}}_{\text{t}}\text{(i)}}{\text{W}_{\text{t}}\text{(i)}}\text{ }\text{×}\text{ }\text{100\%}$ (2)

where W_t_(e) is the weight of the hydrogel after swelling equilibrium state at time interval t and W_t_(i) is the initial weight of the dry hydrogel. Three times measurements were performed for each sample and the average value was used.

**In Vitro Degradation Test**

In vitro degradation behavior of hydrogels was studied using weight loss as a function of time.^[5,6]^ The dried hydrogels were soaked in PBS solution containing 1.6 mg/mL lysozyme and placed in a constant temperature circulating tank at 37 °C. They were removed and weighed after drying on days 7, 14, and 21. The degradation percentage of hydrogel was calculated using the following formula (3):

$\text{Percentage degradation }\left( \text{\%} \right)\text{ }\text{=}\text{ }\frac{\text{W}_{\text{t}}\left( \text{i} \right) \text{-}\text{ }\text{W}_{\text{t}}\left( \text{t} \right)}{\text{W}_{\text{t}}\left( \text{t} \right)}\text{ }\text{×}\text{ }\text{100\%}$ (3)

where W_t_(i) is the initial weight of the dried hydrogel and W_t_(t) is the weight of hydrogel at time interval t. Three times measurements were performed for each sample and the average value was used.

**Cytocompatibility Analysis Cell Culture**

L929 mouse fibroblast cells (ATCC, USA) were cultured in Dulbecco’s Modified Eagle Medium (DMEM, high glucose) supplemented with 10% fetal bovine serum (FBS) and 1% penicillin–streptomycin (Pen/Strep). Cells were maintained in T75 flasks at 37 °C, 5% CO_2_, and 95% humidity, and the culture medium was changed every 2 days. Cells from passage numbers 5–10 were used in all experiments. Metabolic activity was calculated using the following equation:

Metabolic activity (%) = Absorbance of sample / Absorbance of control × 100%

**In Vitro Cytotoxicity Analysis of Hydrogels**

To evaluate the AgNPs-loaded hydrogels cytotoxicity, first, a 10 mm diameter disc (approximately 50 mg) hydrogel was placed in a 48-well plate, then L929 cells were seeded on the hydrogel at a concentration of 5×10^4^ cells and allowed to adhere for 72 hours. The medium was not refreshed during the 72-hour incubation period to maintain consistent exposure conditions for evaluating hydrogel–cell interactions. Biocompatibility of hydrogels was evaluated using the 3-(4,5-dimethylthiazol-2-yl)-2,5-diphenyl tetrazolium bromide (MTT) assay. Cells cultured in a medium without hydrogels were used as the control group. Tests were repeated thrice for each group.

**Antibacterial Analysis**

According to published procedures and some modifications, the method for measuring the antibacterial effect of the hydrogels has been determined.^[7,8]^ To assess the antibacterial effect of the AgNPs-loaded hydrogels, pure cultures of *E. coli* and *S. aureu*s were grown in LB broth at 100 rpm and 37 °C for 15 hours. The absorbance value of the prepared bacterial liquid was controlled at about 0.5 concentration, ​then 50 μL of the bacterial solution was taken out and quickly spread on an agar plate. The plates were inverted and incubated at 37 °C for 24 hours. Finally, the Zone of Inhibition (ZOI) was measured with a ruler. All the tests were performed in triplicates.

**Animal Study**

The experiments were carried out at Taipei Medical University according to the guidelines of care and use of animals. All the protocols for animal study and use were reviewed and approved by the institutional Animal Care and Use Committee of Taipei Medical University under the affidavit no. LAC-2021-0130. We used 5-week-old male Sprague Dawley rats, which were purchased from BioLASCO Taiwan Co., Ltd. (Yilan City, Taiwan). Animals were acclimatized for a minimum of one week prior to experimentation. Animals had ad libitum access to standard chow and water at all times.

**Wound Healing**

The full-thickness rat skin incision model was used to evaluate the effect of hydrogel on wound healing, regarding previous reports and some modifications.^[9,10]^ The experiment was performed using male rats weighing approximately 250 g each. A surgical anesthetic of a 0.25 mL mixture of Zoletil 50 (Virbac, France) and Rompun 20 (Bayer, Germany) at a 1:2 ratio was injected intraperitoneally, and then the dorsal region of rats was shaved for further surgery. Two full-thickness wounds with diameters of 2 cm were made on the back of rats. Treatment of wounds covered by hydrogel materials in rats. The first group was treated by C1N1/AgNPs hydrogel and fixed with Hydrocolloid Thin Dressing (3M, USA) and Transparent Film Dressing Frame Style (3M, USA), the second group was treated by C1N2/AgNPs hydrogel and fixed with Hydrocolloid Thin Dressing and Transparent Film Dressing Frame Style, and the third group was treated without hydrogels and fixed with Hydrocolloid Thin Dressing and Transparent Film Dressing Frame Style as a control group. For the observation of the wound area, the wound area was photographed on the 7^th^ and 14^th^ days. The wound healing area was calculated by Image J software and wound contraction was calculated using the following equation (4):

$\text{Wound contraction }\left( \text{\%} \right)\text{ }\text{=}\text{ }\frac{\text{Area(0)}\text{ }\text{-}\text{ }\text{Area(x)}}{\text{Area(0)}}\text{ }\text{×}\text{ }\text{100\%}$ (4)

where Area(0) is the wound area on the 0 day and Area(x) is the wound area on the x day.

**Histological Observation**

In order to evaluate the epidermal regeneration and inflammation in the wound area with reference to previous reports and some modifications,^[9]^ the rats were euthanized via CO_2_ inhalation and their wound skins were removed and sent to Toson Technology Co., Ltd. for further histological analyses. For each animal, samples with surrounding tissue were carefully removed and stored in 10% buffered formalin, embedded in paraffin, and then cross-sectioned to 3 μm thickness slices. Sections were stained using Hematoxylin and Eosin (H&E), Masson’s Trichrome, and immunofluorescence staining for CD31 (endothelial marker) and CD68 (macrophage marker).

For immunofluorescence, tissue sections were deparaffinized, rehydrated through graded ethanol, and underwent heat-induced antigen retrieval in citrate buffer (pH 6.0). Non-specific binding was blocked with 5% bovine serum albumin (BSA). Sections were incubated overnight at 4 °C with primary antibodies against CD31 and CD68, followed by fluorophore-conjugated secondary antibodies. Nuclei were counterstained with DAPI. All stained slides were analyzed and photographed using a fluorescence microscope. Semi-quantitative image analysis was conducted using ImageJ.

**Statistical Analysis**

All data were analyzed and visualized by Origin 2017 software (USA). All the quantified data were presented as the mean ± standard error of mean (SEM) with the sample size n = 3 (n = 5 for animal studies). For wound healing experiments, data was processed using two way ANOVA and Tukey post-hoc methods.

Supporting Tables

**Table S1.** The photographs of CSPP, C2N1, C1N1, and C1N2 copolymer hydrogels and the photographs of copolymers with AgNPs.

|  | **CSPP** | **C2N1** | **C1N1** | **C1N2** |
| --- | --- | --- | --- | --- |
| **Copolymer** | 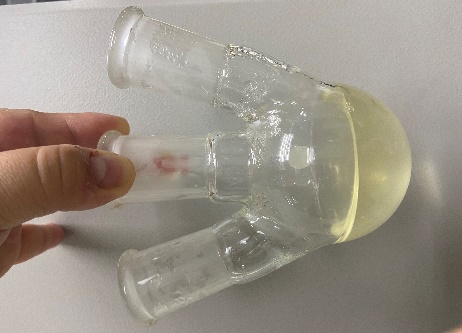 | 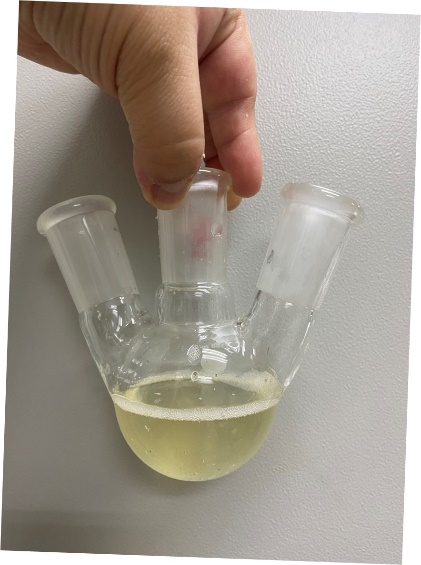 | 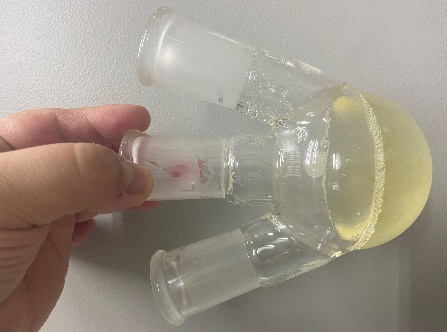 | **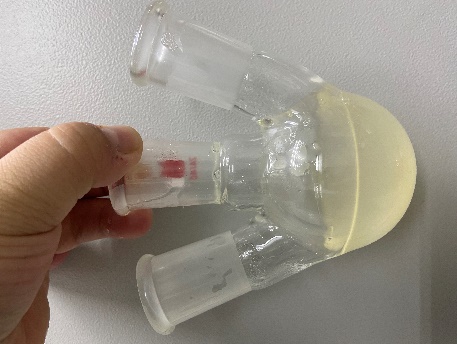** |
| **Hydrogel film** | 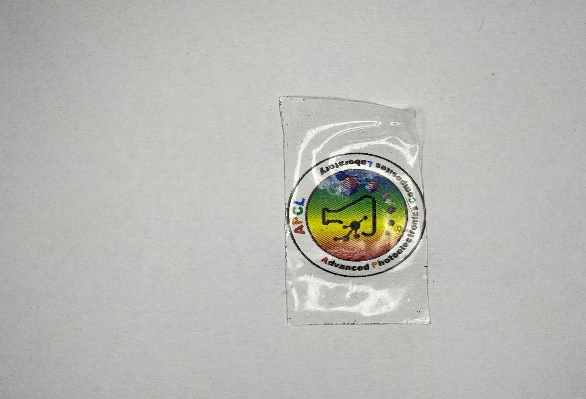 | 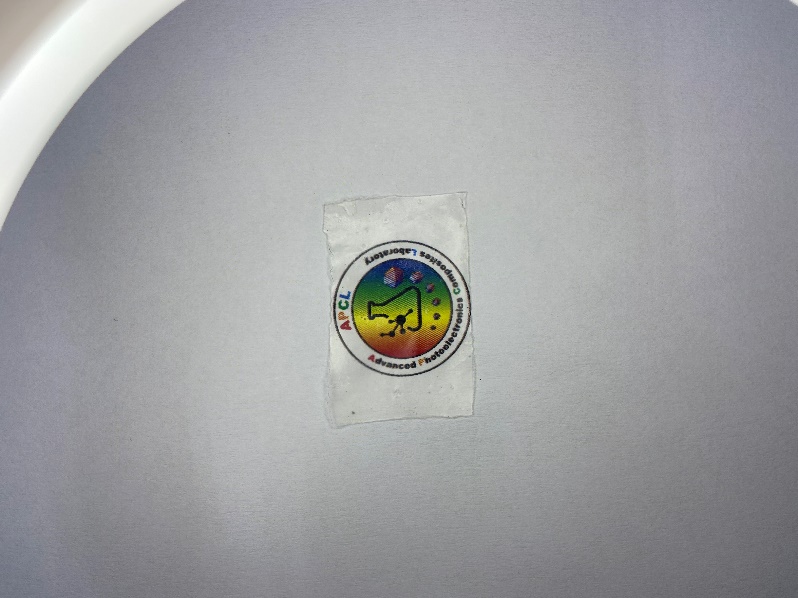 | 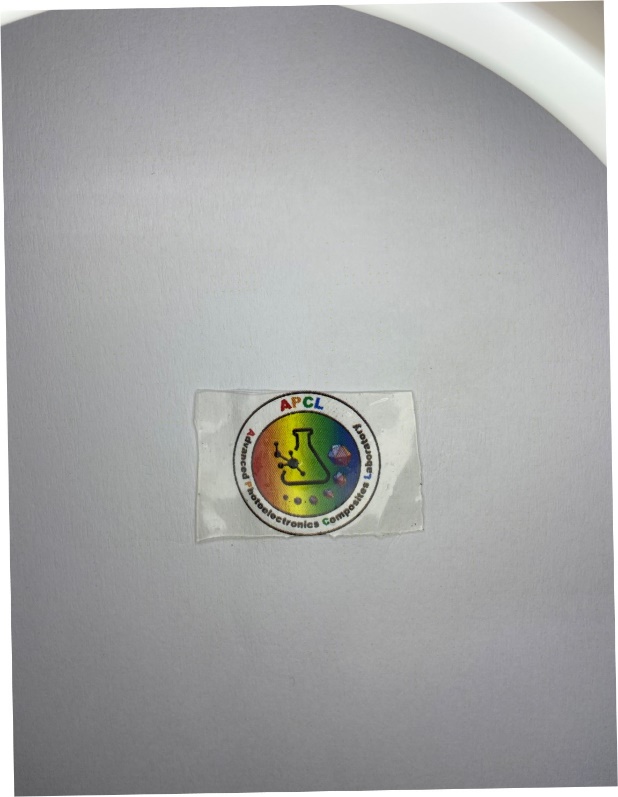 | 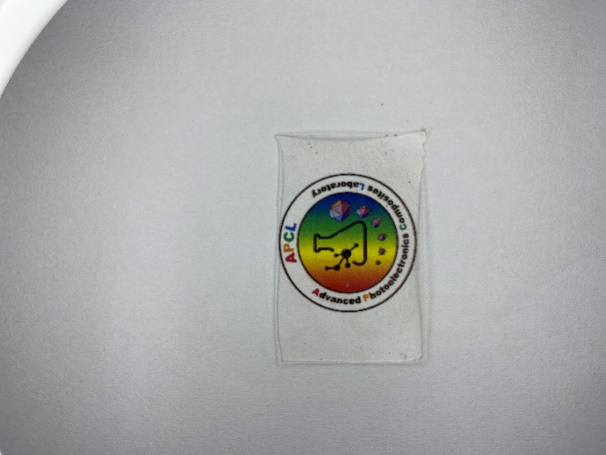 |
| **AgNPs loaded copolymer** | 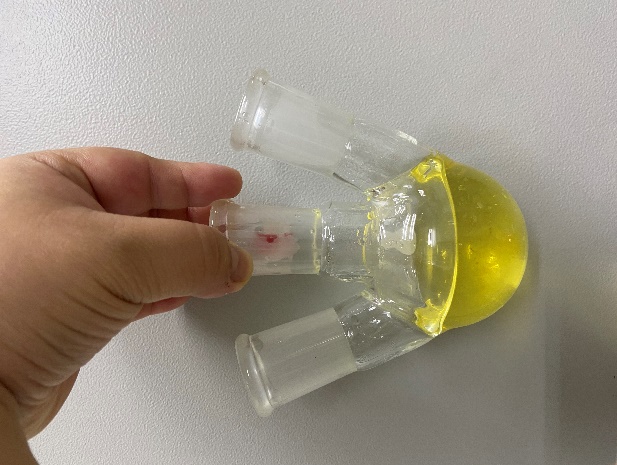 | 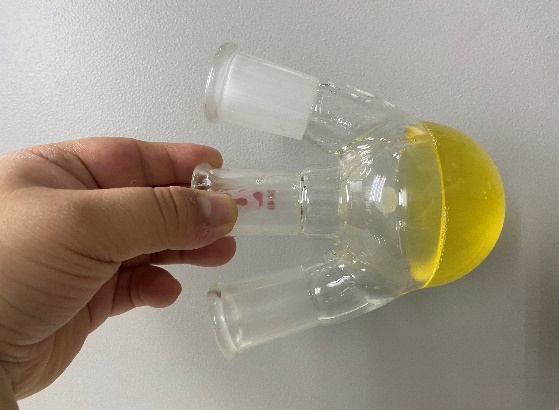 | 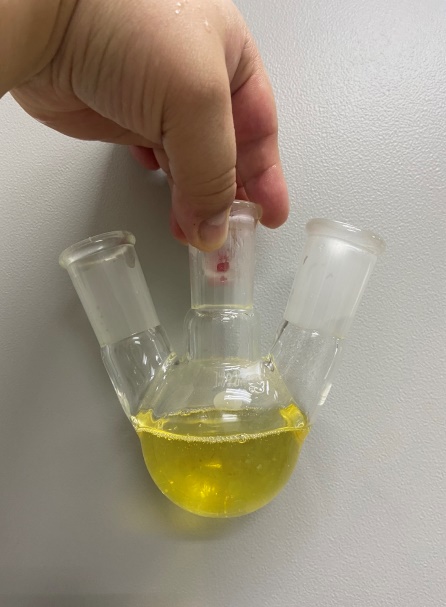 | 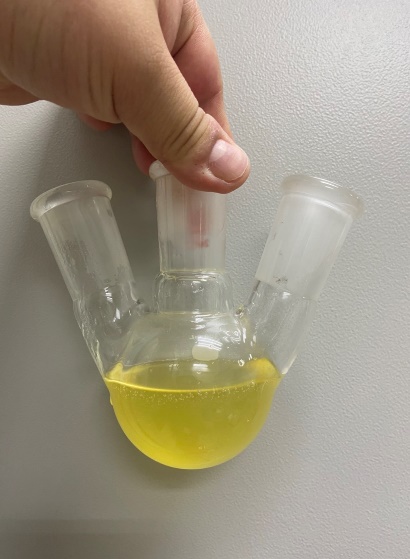 |

**Table S2.** (a) The molecular weigh of DMF-soluble fractions; (b) moisture content; (c) melting point; (d) glass transition temperature; (e) compression strength; and (f) compression strain of CSPP, C2N1, C1N1, and C1N2 hydrogels.

| **Sample** | **(a)**  **Molecular weight** | **(b)**  **Moisture content** | **(c)**  **Tm** | **(d)**  **Tg** | **(e)**  **Strength** | **(f)**  **Strain** |
| --- | --- | --- | --- | --- | --- | --- |
|  | **g/mol** | **%** | $\text{°C}$ | °C | kPa | % |
| **CSPP** | 52,135 | 88.53 | 143.17 | 53.63 | 567.94 | 59.37 |
| **C2N1** | 44,061 | 70.79 | 139.12 | 57.75 | 747.57 | 63.36 |
| **C1N1** | 37,422 | 76.92 | 129.33 | 67.30 | 748.26 | 71.90 |
| **C1N2** | 44,099 | 74.90 | 118.94 | 71.41 | 749.31 | 77.55 |

**Table S3.** The appearance of CSPP, C2N1, C1N1, and C1N2 hydrogels at 25 °C and 40 °C.

| **Temperature** | **CSPP** | **C2N1** | **C1N1** | **C1N2** |
| --- | --- | --- | --- | --- |
| **25**°C | 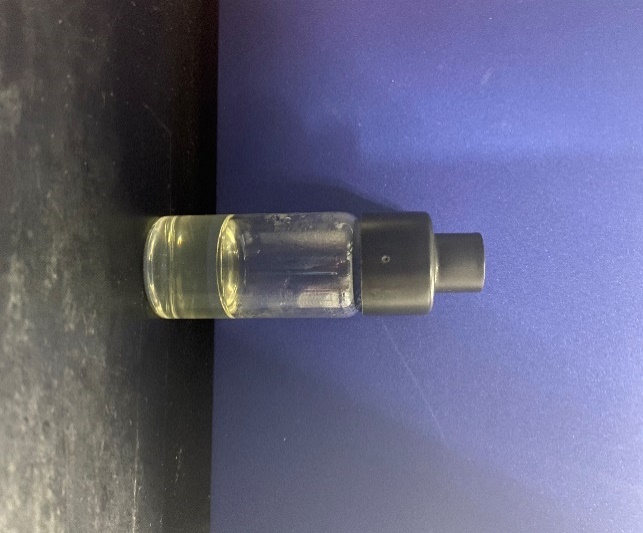 | 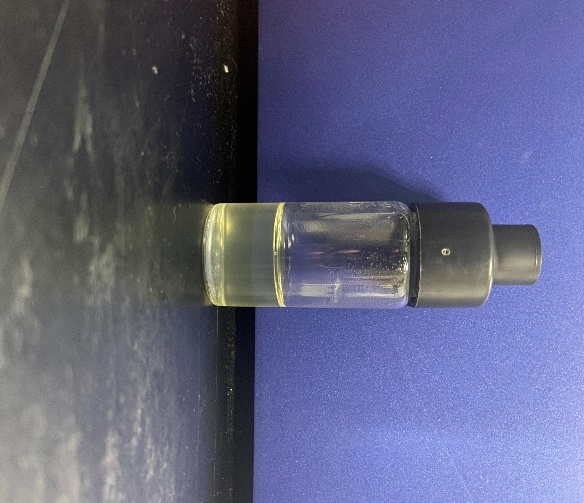 | 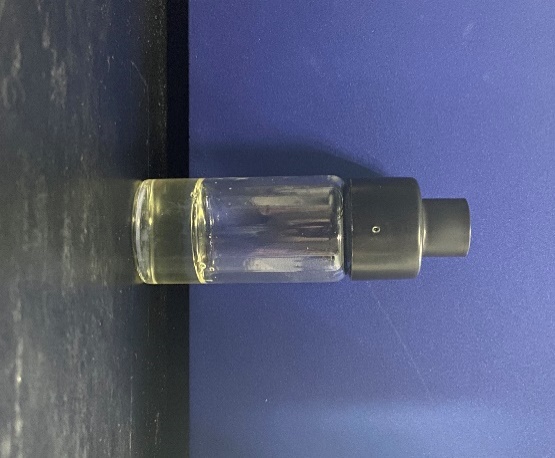 | 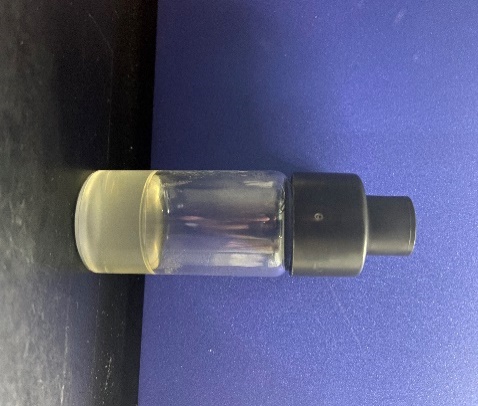 |
| **40**°C | 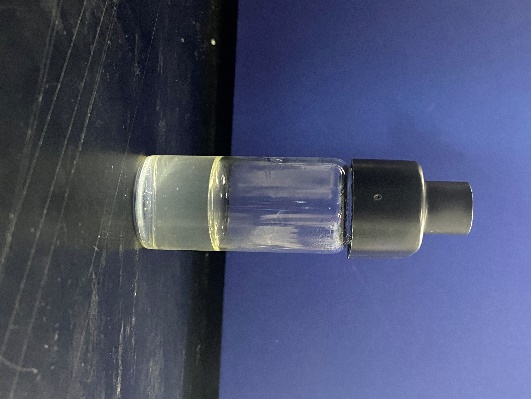 | 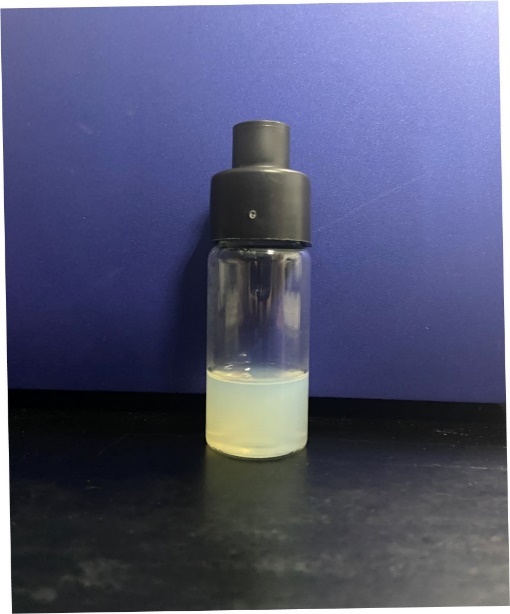 | 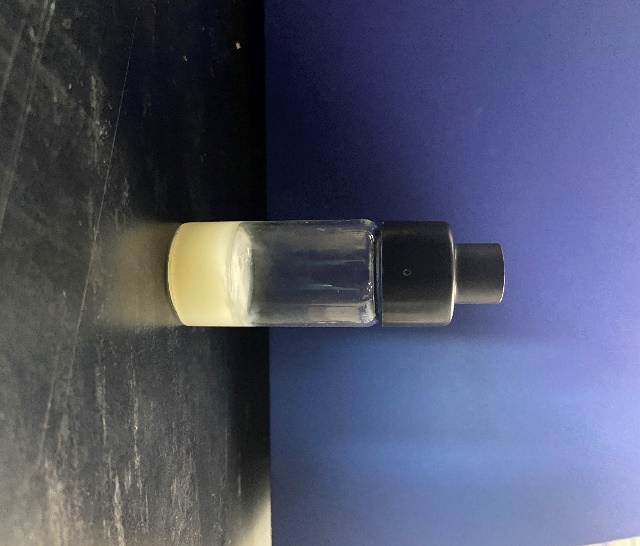 | 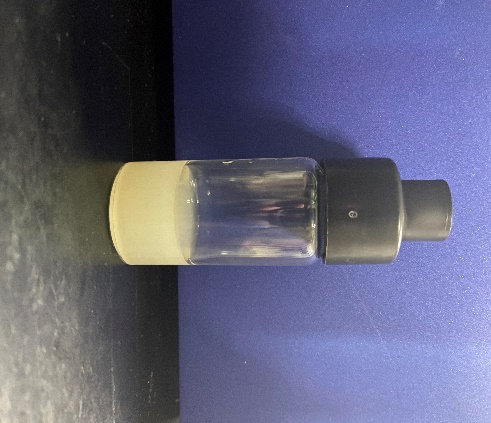 |

**Table S4.** Statistical analysis of wound healing experiment using (a) Two-Way ANOVA and (b) Tukey post-hoc test.

| **a** | **Sample** |  | | **Day 7** | | |  | | **Day 14** | | | | | | ***F* value** | ***p* value** |  |  |
| --- | --- | --- | --- | --- | --- | --- | --- | --- | --- | --- | --- | --- | --- | --- | --- | --- | --- | --- |
|  |  | **Mean** | | **SD** | | **SEM** | | | | **Mean** | **SD** | | | **SEM** |  |  |  |  |
|  | **C1N1** | 82.33 | | 6.85 | | 3.06 | | | | 99.11 | 0.50 | | | 0.22 |  |  |  |  |
|  | **C1N2** | 73.43 | | 17.62 | | 7.88 | | | | 96.69 | 4.20 | | | 1.88 | 4.70 | 0.02 |  |  |
|  | **Control** | 47.97 | | 25.94 | | 11.60 | | | | 97.97 | 0.77 | | | 0.34 |  |  |  |  |
|  | ***F* value** | 38.65 | | | | | | | | | | | | | Interaction | |  |  |
|  | ***p* value** | 2.00 ×10^-6^ | | | | | | | | | | | | | 0.02 | |  |  |
|  |  |  | | | | | | | | | | | | |  | |  |  |
| **b** | | **Interaction** | | | |  | | **Mean Diff.** | | | | | **Significant** | | | | | |
|  |  | **C1N1 day 14** | | **C1N1 day 7** | | | |  | 16.78 | | | | | No | | | | |
|  |  | **C1N2 day 7** | | **C1N1 day 7** | | | |  | -8.90 | | | | | No | | | | |
|  |  | **C1N2 day 7** | | **C1N1 day 14** | | | |  | -25.68 | | | | | No | | | | |
|  |  | **C1N2 day 14** | | **C1N1 day 7** | | | |  | 14.357 | | | | | No | | | | |
|  |  | **C1N2 day 14** | | **C1N1 day 14** | | | |  | -2.43 | | | | | No | | | | |
|  |  | **C1N2 day 14** | | **C1N2 day 7** | | | |  | 23.25 | | | | | No | | | | |
|  |  | **Control day 7** | | **C1N1 day 7** | | | |  | -34.36 | | | | | Yes | | | | |
|  |  | **Control day 7** | | **C1N1 day 14** | | | |  | -51.14 | | | | | Yes | | | | |
|  |  | **Control day 7** | | **C1N2 day 7** | | | |  | -25.46 | | | | | No | | | | |
|  |  | **Control day 7** | | **C1N2 day 14** | | | |  | -48.72 | | | | | Yes | | | | |
|  |  | **Control day 14** | | **C1N1 day 7** | | | |  | 15.64 | | | | | No | | | | |
|  |  | **Control day 14** | | **C1N1day 14** | | | |  | -1.14 | | | | | No | | | | |
|  |  | **Control day 14** | | **C1N2 day 7** | | | |  | 24.54 | | | | | No | | | | |
|  |  | **Control day 14** | | **C1N2 day 14** | | | |  | 1.28 | | | | | No | | | | |
|  |  | **Control day 14** | | **Control day 7** | | | |  | 50.00 | | | | | Yes | | | | |

Supporting Figures


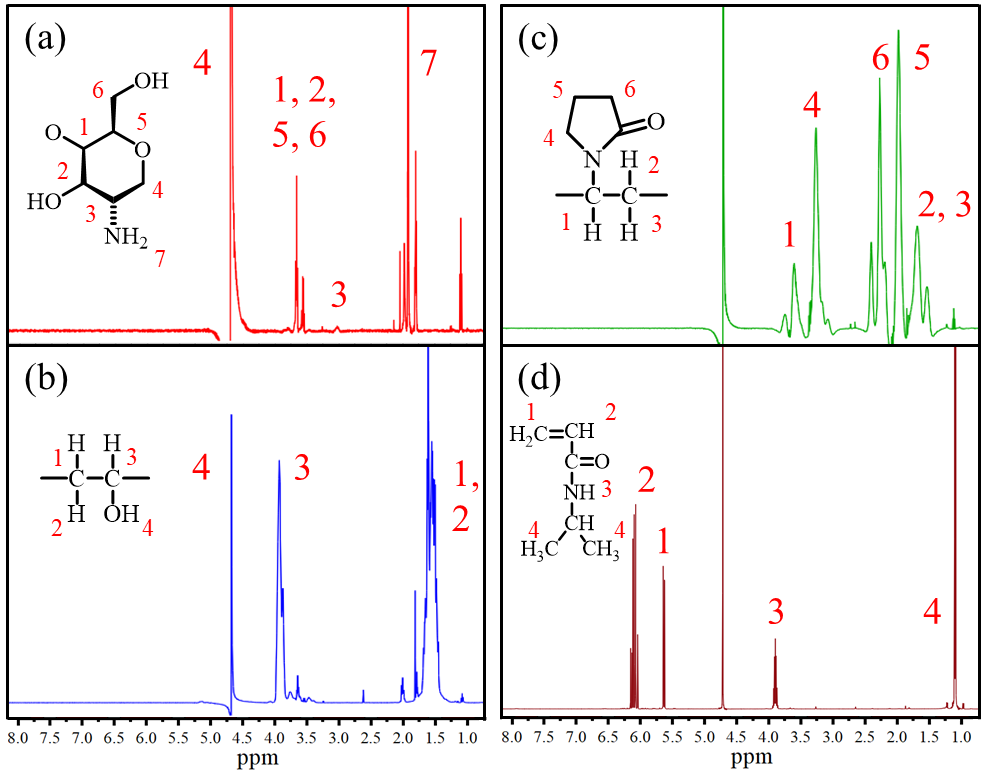


**Figure S1.** NMR spectrum of hydrogel raw material monomer. (a) Chitosan; (b) PVA; (c) PVP; (d) NIPAAm.

The solvent used in all samples is D_2_O, and its chemical shift is 4.7 ppm. **Figure S1(a)** is the ^1^H NMR structure analysis of chitosan. Its characteristic peaks are as follows: δ_4_ is the hydrogen of the C-CH_2_-O group on the six-membered ring of sugar. δ_1, 2, 5, 6_ are hydrogen on the C-CH-C group. δ_3_ is the hydrogen near the CH group of the primary amine. δ_7_ is hydrogen on NH_2_. **Figure S1(b)** is the ^1^H NMR structural analysis of PVA. Its characteristic peaks are as follows: δ_4_ is the hydrogen on the C-OH group. δ_3_ is the hydrogen of the O-C-CH group. δ_1,2_ is the hydrogen of the C-CH_2_ group. **Figure S1(c)** is the ^1^H NMR structural analysis of PVP. Its characteristic peaks are as follows: δ_1_ is the hydrogen near the CH group of amide. δ_4_ is the hydrogen of the CH group beside γ-lactam. δ_6_ is the hydrogen of the CH group on the diagonal of γ-lactam. δ_5_ is the hydrogen of the CH group close to C=O on the γ-lactam. δ_2, 3_ is the hydrogen of the C-CH_2_ group. **Figure S1(d)** is the ^1^H NMR structure analysis of NIPAAm. In the region between 5.5–6.5 ppm, there are signals of hydrogen adjacent to the double-bonded carbon, and these peaks are clearly visible in the NMR spectrum. δ_2_ is the hydrogen near the C-CH=C group of amides. δ_1_ is the hydrogen of the C=CH_2_ group. δ_3_ is the hydrogen of the CH group beside the amide. δ_4_ is the hydrogen of the C-CH_3_ group.


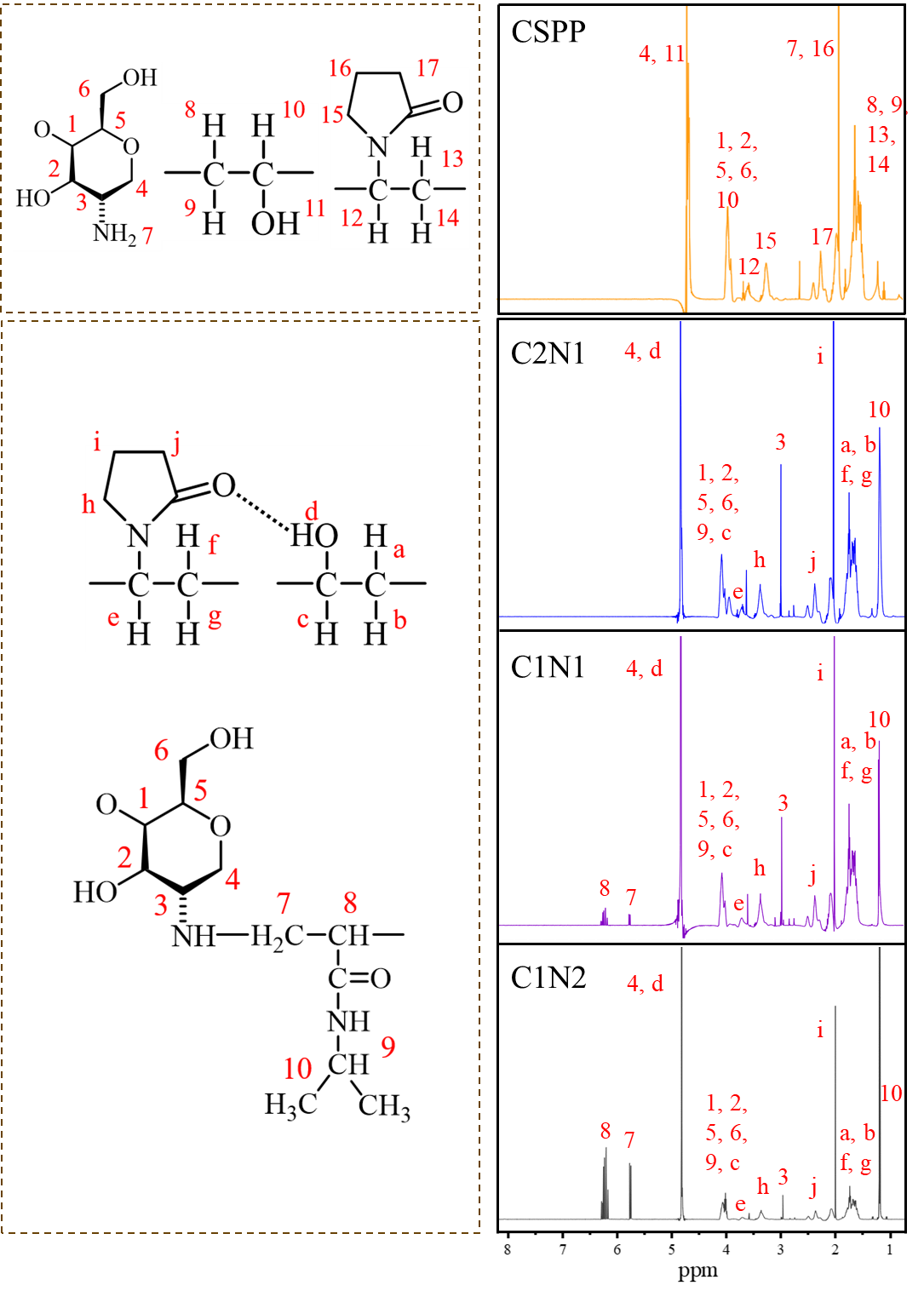


**Figure S2.** ^1^H NMR spectrum of all prepared samples (CSPP, C2N1, C1N1, and C1N1) for confirmation of synthesis success.


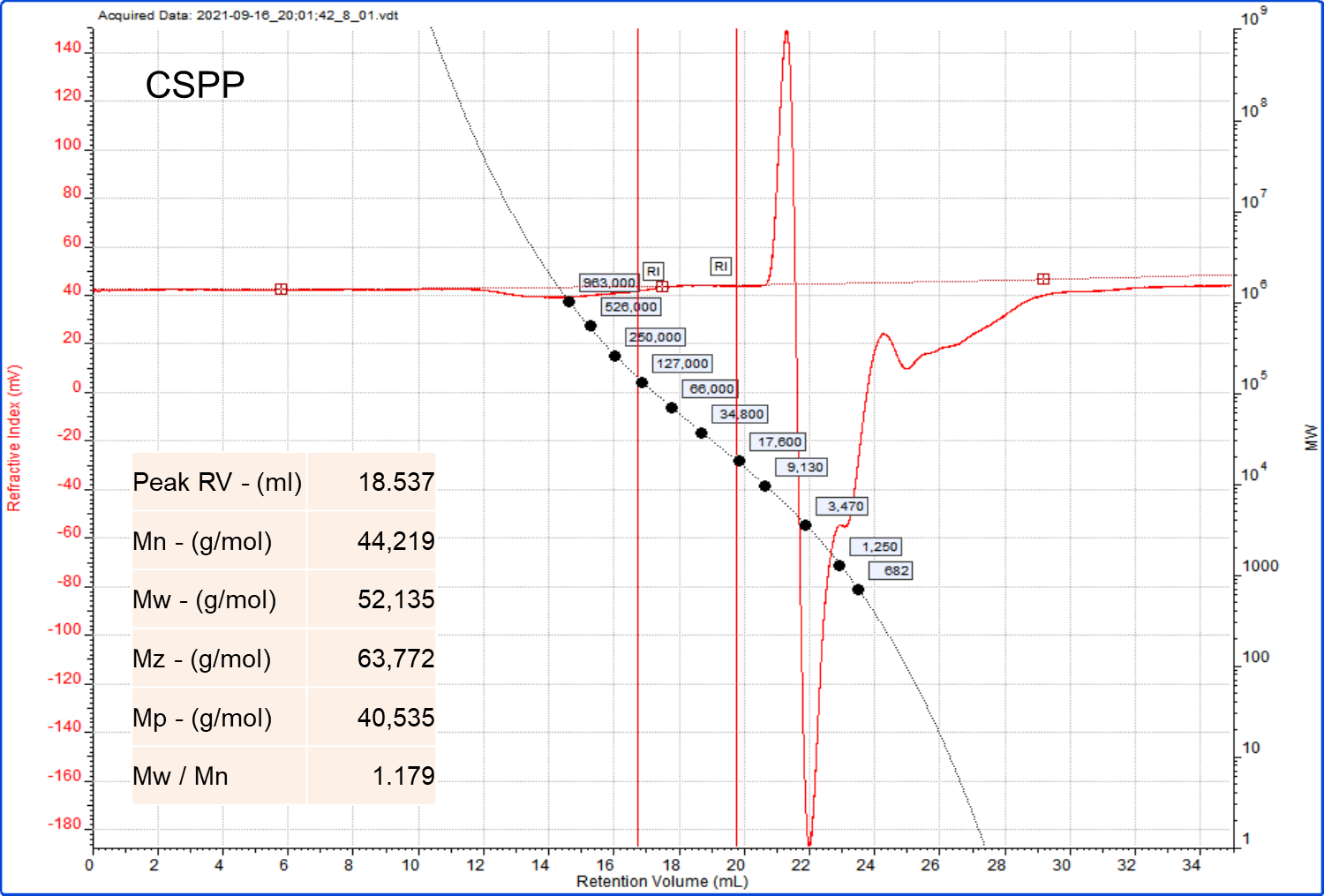


**Figure S3.** GPC chromatogram of CSPP hydrogel sample.


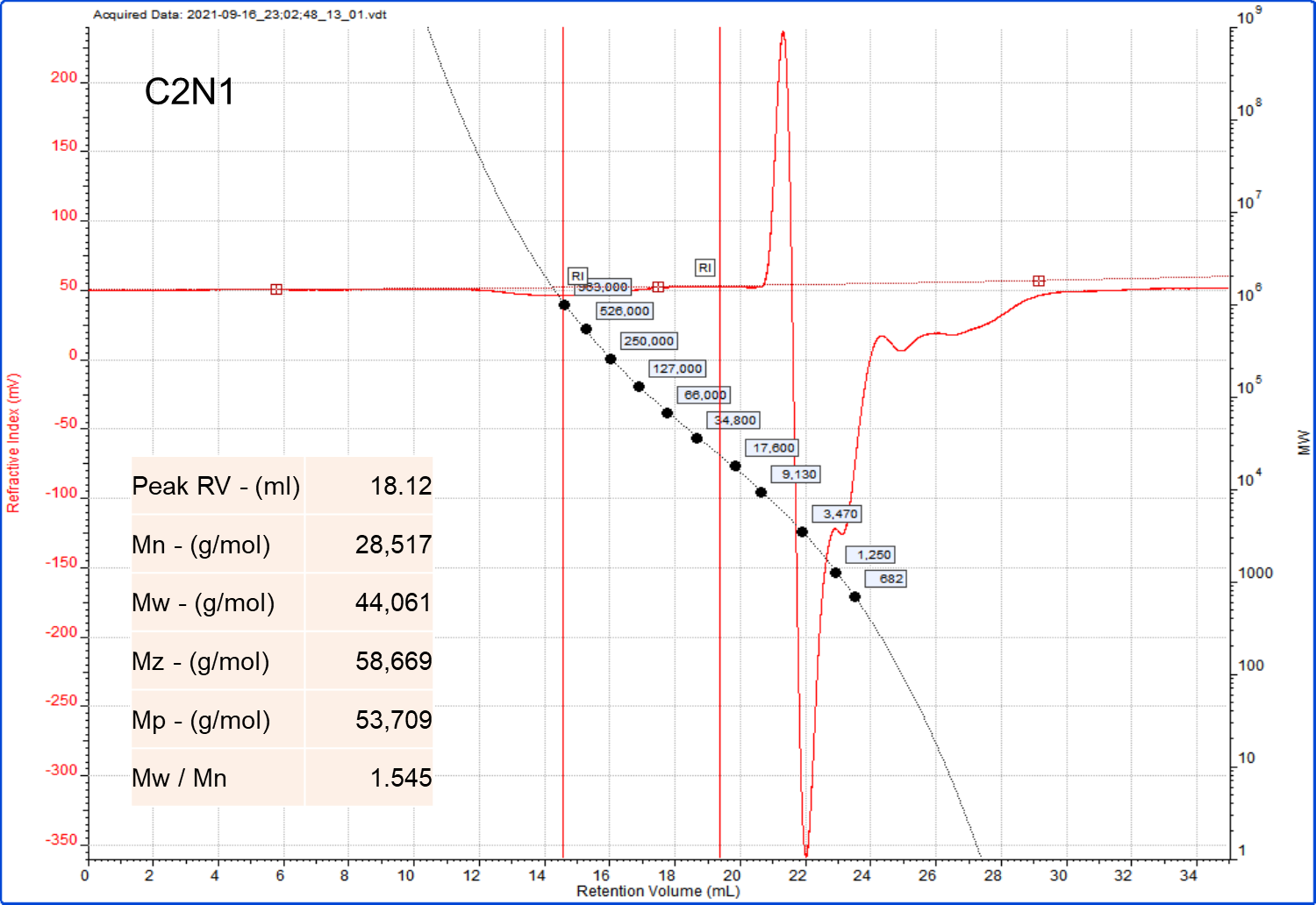


**Figure S4.** GPC chromatogram of C2N1 hydrogel sample.


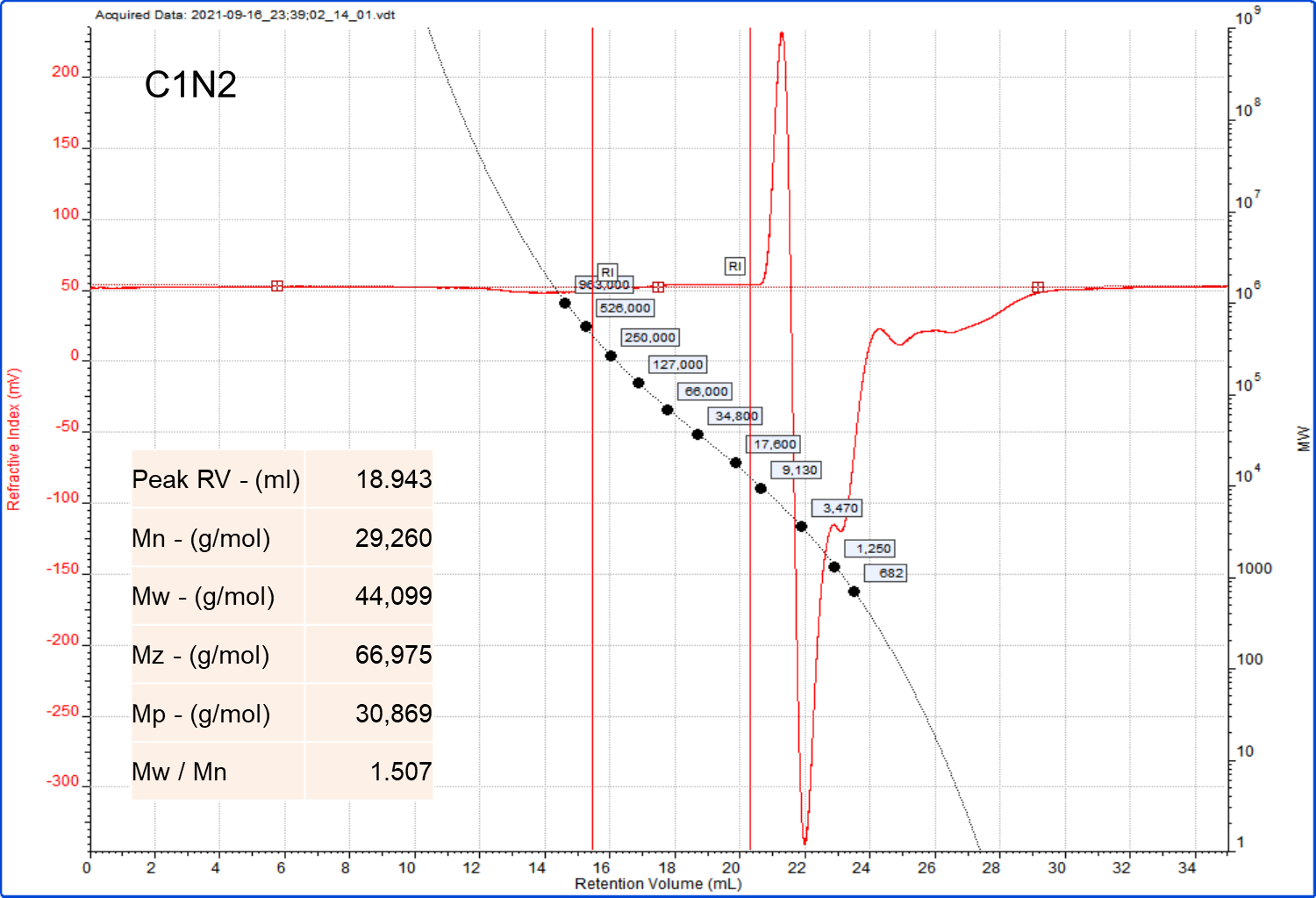


**Figure S5.** GPC chromatogram of C1N2 hydrogel sample.


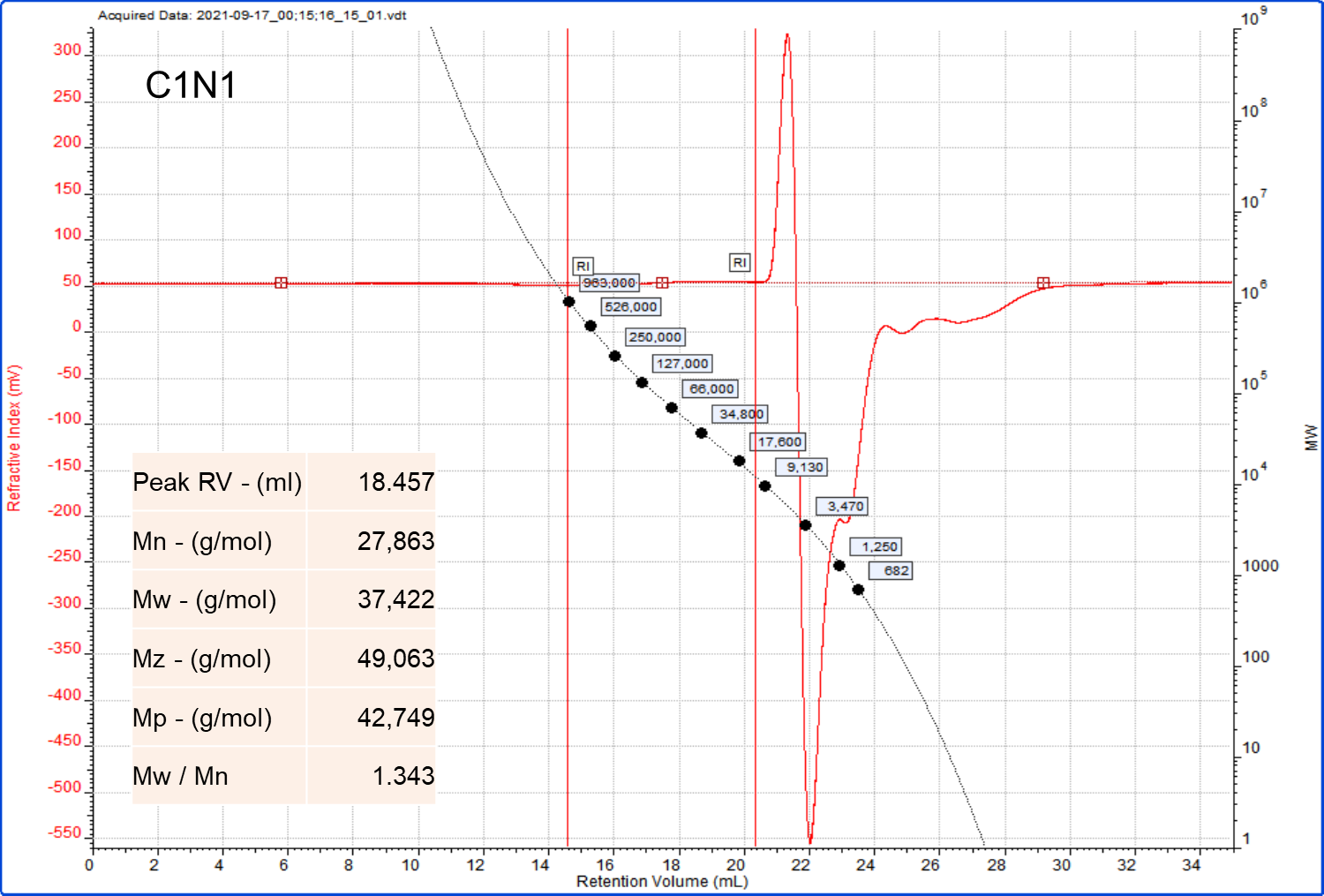


**Figure S6.** GPC chromatogram of C1N1 hydrogel sample.


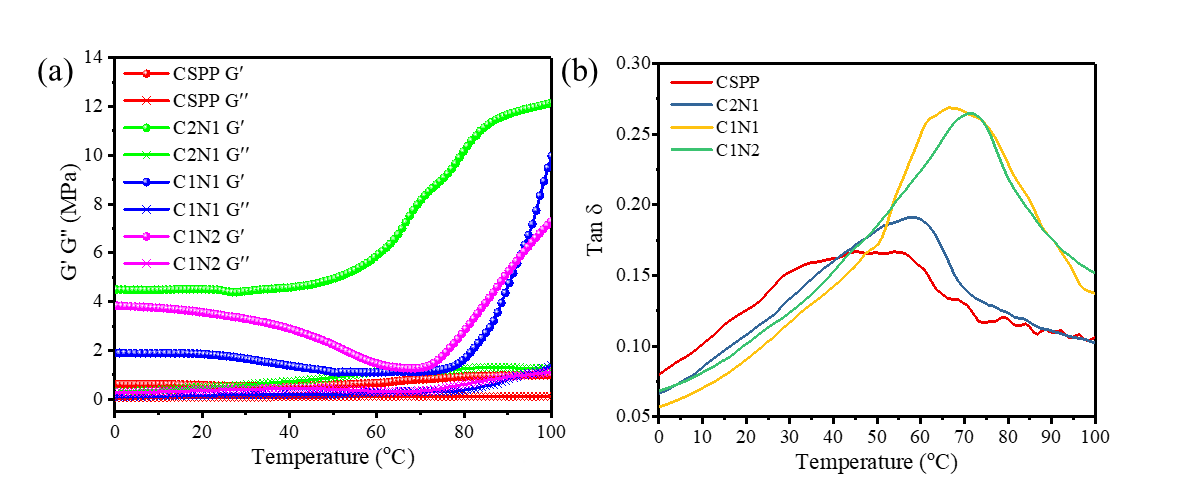


**Figure S7.** (a) Trends in storage and loss moduli of all samples as a function of temperature. (b) Calculated ratio of loss modulus to storage modulus to determin Tg.


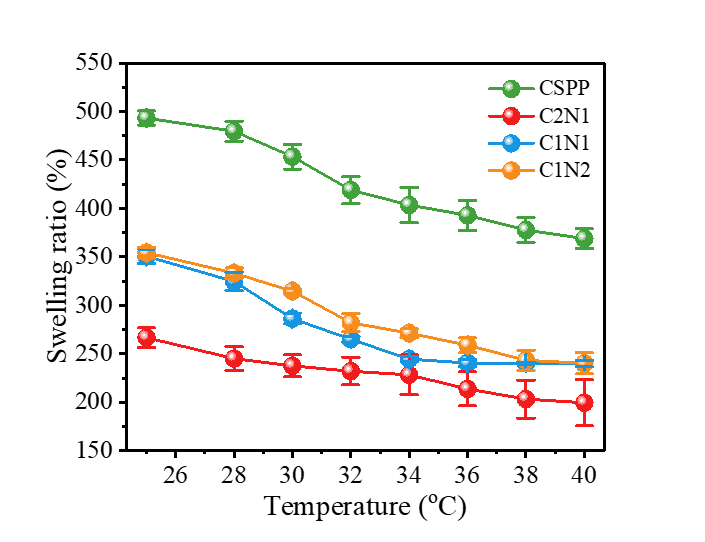


**Figure S8.** Temperature dependences of the swelling ratio curve for hydrogels (mean ± SEM, n = 3).


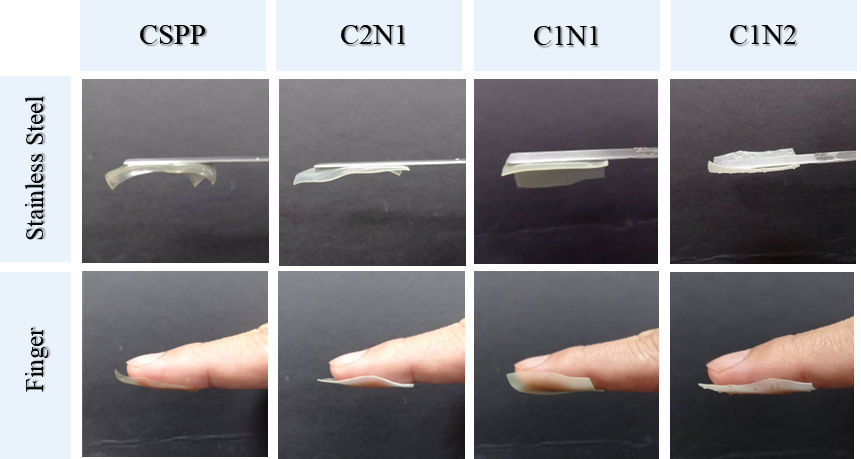


**Figure S9.** Adhesivity of the prepared hydrogels.


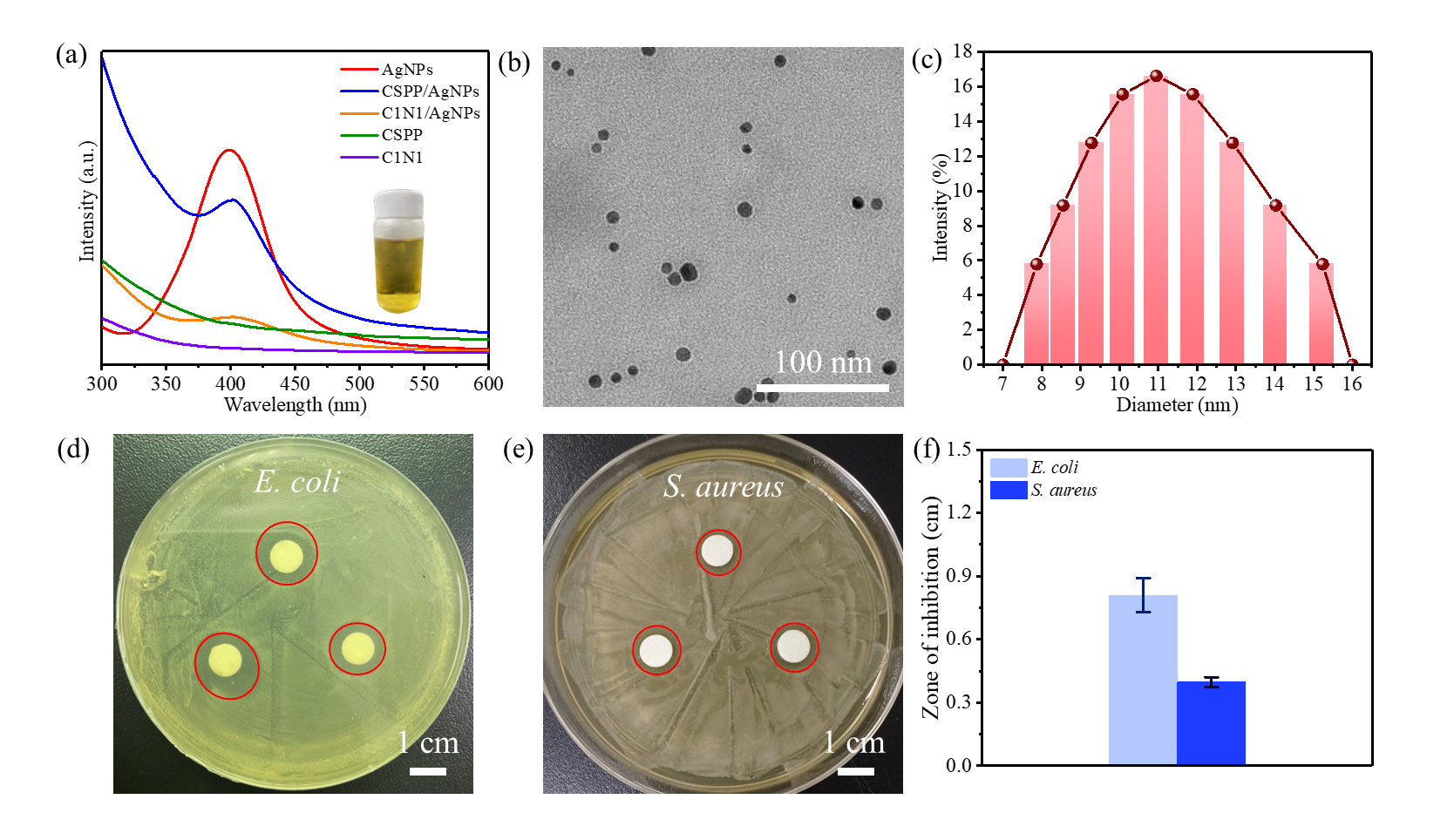


**Figure S10.** Analysis of AgNPs. (a) UV spectra of AgNPs and hydrogels; (b) Morphology of AgNPs under TEM; (c) Size distribution of AgNPs; (d-f) Antibacterial effect of AgNPs without hydrogel medium (used filter paper as medium) over *E. coli* and *S. aureus*.


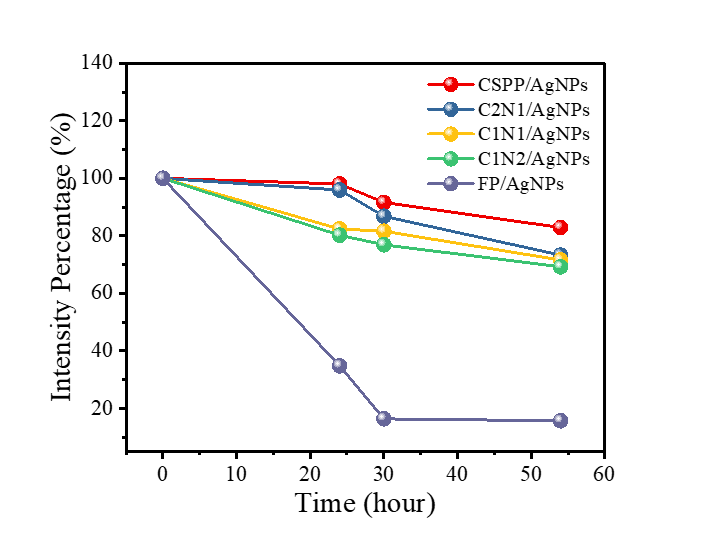


**Figure S11.** Time-dependent intensity comparison of the AgNPs corresponding peak (Figure S10a) for the sample immersed in water (control FP = using filter paper).


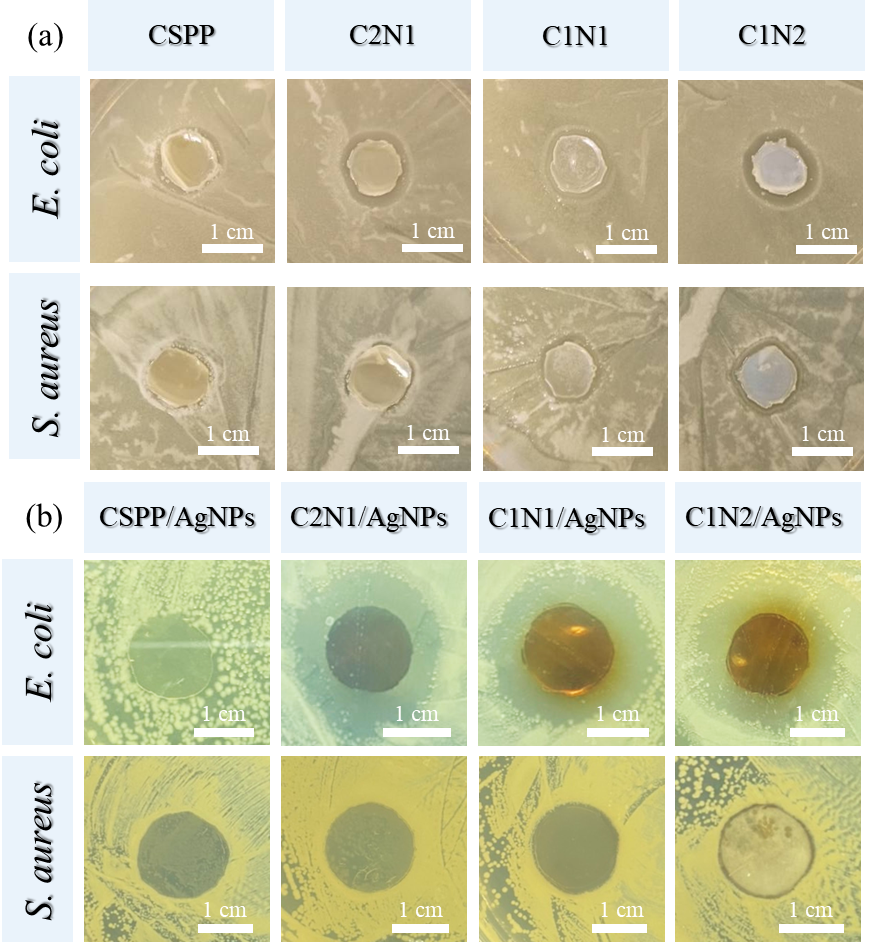


**Figure S12.** The antibacterial effect of (a) hydrogels (CSPP, C2N1, C1N1, and C1N2) and (b) AgNPs-loaded hydrogels againts *E. coli* and *S. aureus* after 24 hours.


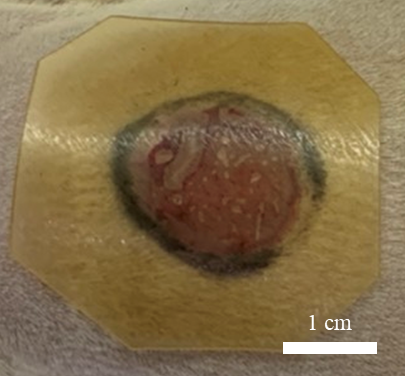


**Figure S13.** Hydrogel attachment on the wound on the rat.

References

[1] S. Agnihotri, S. Mukherji, S. Mukherji, Size–controlled silver nanoparticles synthesized over the range 5–100 nm using the same protocol and their antibacterial efficacy, RSC Adv. 4 (**2014**) 3974–3983.

[2] M. Alboofetileh, M. Rezaei, H. Hosseini, M. Abdollahi, Effect of montmorillonite clay and biopolymer concentration on the physical and mechanical properties of alginate nanocomposite films, J. Food Eng. 117 (**2013**) 26–33.

[3] S. Naghdi, M. Rezaei, M. Abdollahi, A starch-based pH-sensing and ammonia detector film containing betacyanin of paperflower for application in intelligent packaging of fish, Int. J. Biol. Macromol. 191 (**2021**) 161–170.

[4] M. Ghaemy, M. Naseri, Synthesis of chitosan networks: Swelling, drug release, and magnetically assisted BSA separation using Fe_3_O_4_ nanoparticles, Carbohydr. Polym. 90 (**2012**) 1265–1272.

[5] A. Kumar, T. Behl, S. Chadha, Synthesis of physically crosslinked PVA/Chitosan loaded silver nanoparticles hydrogels with tunable mechanical properties and antibacterial effects, Int. J. Biol. Macromol. 149 (**2020**) 1262–1274.

[6] B.B. Mandal, S. Kapoor, S.C. Kundu, Silk fibroin/polyacrylamide semi-interpenetrating network hydrogels for controlled drug release, Biomaterials 30 (**2009**) 2826–2836.

[7] Y. Xie, X. Liao, J. Zhang, F. Yang, Z. Fan, Novel chitosan hydrogels reinforced by silver nanoparticles with ultrahigh mechanical and high antibacterial properties for accelerating wound healing, Int. J. Biol. Macromol. 119 (**2018**) 402–412.

[8] T.W. Kang, G. Chandrasekaran, E.C. Hwang, H.S. Kim, V.-K. Lakshmanan, Characterization and antibacterial activity of PVA–PVP–CS carvacrol-loaded polymer composite films for urinary catheter, Int. J. Polym. Mater. Polym. Bio. 67 (**2018**) 1016–1027.

[9] X. Zhao, Y. Liang, Y. Huang, J. He, Y. Han, B. Guo, Physical double network hydrogel adhesives with rapid shape adaptability, fast self-healing, antioxidant and NIR/pH stimulus responsiveness for multidrug resistant bacterial infection and removable wound dressing, Adv. Funct. Mater. 30 (**2020**) 1910748–1910765.

[10] X. Liu, W.C. Liu, H.Y. Wang, V.L. Li, Y.C. Chen, A.N. Wang, C.J. Wu, Y. Li, G. Zhao, C. Lin, A.K. Panda, M. Keerthi, R.J. Chung, Polyelectrolyte multilayer composite coating on 316 L stainless steel for controlled release of dual growth factors accelerating restoration of bone defects, Mater. Sci. Eng. C 126 (**2021**) 112187–112198.
